# Foot-and-Mouth Disease Virus Counteracts on Internal Ribosome Entry Site Suppression by G3BP1 and Inhibits G3BP1-Mediated Stress Granule Assembly *via* Post-Translational Mechanisms

**DOI:** 10.3389/fimmu.2018.01142

**Published:** 2018-05-25

**Authors:** Xu Ye, Ting Pan, Dang Wang, Liurong Fang, Jun Ma, Xinyu Zhu, Yanling Shi, Keshan Zhang, Haixue Zheng, Huanchun Chen, Kui Li, Shaobo Xiao

**Affiliations:** ^1^State Key Laboratory of Agricultural Microbiology, College of Veterinary Medicine, Huazhong Agricultural University, Wuhan, China; ^2^The Cooperative Innovation Center for Sustainable Pig Production, Wuhan, China; ^3^National Foot and Mouth Diseases Reference Laboratory, Lanzhou Veterinary Research Institute, Chinese Academy of Agricultural Sciences, Lanzhou, China; ^4^Department of Microbiology, Immunology and Biochemistry, University of Tennessee Health Science Center, Memphis, TN, United States

**Keywords:** foot-and-mouth disease virus, phosphoproteomics, G3BP stress granule assembly factor 1, internal ribosome entry site, innate immunity

## Abstract

Foot-and-mouth disease (FMD) is a highly contagious, severe viral illness notifiable to the World Organization for Animal Health. The causative agent, FMD virus (FMDV), replicates rapidly and efficiently inhibits host translation and the innate immune response for it has developed multiple tactics to evade host defenses and takes over gene expression machinery in the host cell. Here, we report a systemic analysis of the proteome and phosphoproteome of FMDV-infected cells. Bioinformatics analysis suggested that FMDV infection shuts off host cap-dependent translation, but leaves intact internal ribosome entry site (IRES)-mediated translation for viral proteins. Interestingly, several FMDV IRES-transacting factors, including G3BP stress granule assembly factor 1 (G3BP1), were dephosphorylated during FMDV infection. Ectopic expression of G3BP1 inhibited FMDV IRES activity, promoted assembly of stress granules, and activated innate immune responses, collectively suppressing FMDV replication. To counteract these host protective responses, FMDV-induced dephosphorylation of G3BP1, compromising its inhibitory effect on viral IRES. In addition, FMDV also proteolytically cleaved G3BP1 by its 3C protease (3C^pro^). G3BP1 was cleaved at glutamic acid-284 (E284) by FMDV 3C^pro^, and this cleavage completely lost the abilities of G3BP1 to activate innate immunity and to inhibit FMDV replication. Together, these data provide new insights into the post-translational mechanisms by which FMDV limits host stress and antiviral responses and indicate that G3BP1 dephosphorylation and its proteolysis by viral protease are important factors in the failure of host defense against FMDV infection.

## Introduction

Foot-and-mouth disease (FMD) severely compromises livestock production, ensuing high economic losses, and international restrictions on the export of animals and animal products (1). The etiologic agent, FMD virus (FMDV), is a positive-stranded RNA virus that belongs to the *Aphthovirus* genus of the *Picornaviridae* family. FMDV genome consists of a single-stranded RNA genome of 8.5 kb that encodes a large polyprotein translated under control of an internal ribosome entry site (IRES) located in the 5’ untranslated region (2). During nearly all picornavirus infections, cellular cap-dependent translation is shutdown, giving way to IRES-mediated translation of viral RNAs that synthesizes the viral polyprotein (3). Upon cleavage by two virus-encoded proteinases, i.e., leader (L^pro^) and 3C protease (3C^pro^), the FMDV polyprotein is processed into intermediate precursors and mature, individual structural and nonstructural proteins that implement diverse functions in viral life cycle (2). To propagate rapidly and efficiently at initial site of infection, FMDV has evolved multi-pronged strategies to regulate cellular gene expression, stress responses, and host innate immune responses (4–6). Although significant progress has been made in recent years in identifying the vital players in both the host and the pathogen, the complex interactions between FMDV and host cell, and the underlying molecular mechanisms, remain to be elucidated.

Proteomics can shed light on the complex and dynamic response of the host to pathogens, and provide a detailed understanding of disease mechanisms based on the system-level information of the host response (7–10). Thus, a powerful tool namely “mass spectrometry-based proteomics” was beneficial for analyzing protein abundances, modifications, and interactions. Accumulating evidence suggests that hijacking host post-translational mechanisms such as protein phosphorylation is a crucial strategy for viruses to proficiently destabilize host signal transduction pathways (10). Current advances in quantitative phosphoproteomics have made it conceivable to profile the changes in host protein phosphorylation during virus infections (7–9). To the best of our knowledge, no such analysis has been testified yet in a picornavirus infection setting. Obviously, a comprehensive, system-level quantitative phosphoproteome analysis is desired to understand how and to what extent FMDV takeovers host cell phosphorylation to cause diseases.

In this study, a stable isotope labeling by amino acids in cell culture (SILAC)-based quantitative phosphoproteomics approach was employed to assess the impact of FMDV infection on host cell phosphoproteome. We identified 2,671 porcine phosphoproteins and in total 884 proteins showed changes in their phosphorylation status upon FMDV infection, suggesting that the FMDV infection has a profound effect on host protein phosphorylation. Additional infection studies centered on a newly identified host target, G3BP stress granule assembly factor 1 (G3BP1), as a novel inhibitor of FMDV IRES-dependent translation, illustrating an unappreciated mechanism of suppressing FMDV replication by G3BP1. FMDV, in turn, promoted dephosphorylation of G3BP1 and targeted this host factor for proteolysis by its 3C^pro^, thereby relieving the inhibitory effort on viral IRES and disrupting stress granule (SG) formation and innate immune activation. Together, our study reveals that dephosphorylation and proteolytic cleavage of G3BP1 are two new post-translational mechanisms by which FMDV counteracts host stress and innate antiviral responses.

## Materials and Methods

### Sample Preparation for Phosphoproteome Analysis

SILAC-labeling of porcine kidney cells (IBRS-2) was performed in lysine- and arginine-free DMEM (Gibco) supplemented with 10% (v/v) dialyzed FBS together with unlabeled lysine and arginine [“light” label (L), mock-infection] or lysine 13C6 and arginine 13C6, 15N4 [“heavy” label (H), FMDV infection, MOI = 5]. At 6 h post-infection (hpi), IBRS-2 cells were washed three times with ice-cold phosphate-buffered saline (PBS) and harvested on ice. Cell lysis was performed similarly to what has been previously described. Briefly, the harvested “heavy” and “light” labeled cells were lysed with lysis buffer supplemented with Phosphatase Inhibitor Cocktail Set II and Protease Inhibitor Cocktail Set IV on ice using a high intensity ultrasonic processor (Scientz) for 30 min, respectively. After mixing heavy and light labeled proteins (1:1), trypsin (Promega) was added into protein solution with ratio of trypsin to protein at 1:50 (w/w) for digestion at 37°C for 16 h. The sample was then fractionated into fractions by high pH reverse-phase high-performance liquid chromatography (HPLC) using Agilent 300Extend C18 column. Peptide mixtures were first incubated with Ti4 + IMAC microspheres suspension with vibration. The supernatant containing phosphopeptides was collected and lyophilized for LC-MS/MS analysis.

### LC-MS/MS Analysis

Peptides were dissolved in solvent A (0.1% formic acid in 2% acetonitrile), directly loaded onto a reversed-phase pre-column (Acclaim PepMap 100, Thermo Scientific). Peptide separation was performed using a reversed-phase analytical column (Acclaim PepMap RSLC, Thermo Scientific) with a linear gradient of 2–20% solvent B (0.1% formic acid in 98% acetonitrile) for 38 min, 20–35% solvent B for 5 min, and 35–80% solvent B for 2 min at a constant flow rate of 300 nl/min on an EASY-nLC 1000 UPLC system. The resulting peptides were analyzed by Q ExactiveTM plus hybrid quadrupole-Orbitrap mass spectrometer (ThermoFisher Scientific).

The peptides were subjected to NSI source followed by MS/MS in Q ExactiveTM plus (Thermo) coupled online to the UPLC. Intact peptides were detected in the Orbitrap at a resolution of 70,000. Peptides were selected for MS/MS using NCE setting as 28; ion fragments were detected in the Orbitrap at a resolution of 17,500. A data-dependent procedure that alternated between one MS scan followed by 20 MS/MS scans was applied for the top 20 precursor ions above a threshold ion count of 1.5E4 in the MS survey scan with 10.0 s dynamic exclusion. The electrospray voltage applied was 2.0 kV. Automatic gain control was used to prevent overfilling of the ion trap; 5E4 ions were accumulated for generation of MS/MS spectra. For MS scans, the *m*/*z* scan range was 350–1,800. Duplicate MS analysis was performed.

### Proteome and Phosphoproteome Date Analysis

The resulting MS/MS data were processed using MaxQuant with integrated Andromeda search engine (v.1.4.1.2). Tandem mass spectra were searched against UniProt_Sus scrofa (26,054 sequences) database concatenated with reverse decoy database. Trypsin/P was specified as cleavage enzyme allowing up to 2 missing cleavages, 5 modifications per peptide, and 5 charges. Mass error was set to 10 ppm for precursor ions and 0.02 Da for fragment ions. Carbamidomethylation on Cys was specified as fixed modification and oxidation on Met, phosphorylation on Ser, Thr, Tyr, and acetylation on protein N-terminal were specified as variable modifications. False discovery rate thresholds for protein, peptide, and modification site were specified at 1%. Minimum peptide length was set at 7. All the other parameters in MaxQuant were set to default values. The site localization probability was set as >0.5. Pathway analysis and classification of proteins based on gene ontology annotations were carried out with IPA (Qiagen, Hilden, Germany).

### RNA Extraction and Quantitative Real-Time Reverse Transcriptase (RT)-PCR

IBRS-2 cells were cultured in 24-well plate and transfected with the indicated plasmids. 30 h later, cells were mock-transfected or infected with FMDV. Total RNA was extracted from the cells using TRIzol reagent (Invitrogen, USA). One microgram of this total RNA was reverse transcribed to cDNA using AMV reverse transcriptase (Toyobo, Japen), which (1 µl of 20 µl cDNA) was subsequently used in a SYBR green PCR assay (Applied Biosystems, USA). The abundance of individual mRNA transcript in each sample was assayed three times and normalized to that of porcine glyceraldehyde-3-phosphate dehydrogenase (GAPDH) mRNA (as an internal control). The primers were designed with Primer Express software v.3.0 (Applied Biosystems, USA).

### Cells, Virus, and Chemicals

Porcine kidney cells (IBRS-2, ATCC number CRL-1835) were cultured in Eagles MEM supplemented with 10% heat-inactivated fetal bovine serum (FBS, Invitrogen), 100 U/ml penicillin and 10 µg/ml streptomycin sulfates, at 37°C with 5% CO_2_ in a humidified incubator. FMDV (strain O/ES/2001, GenBank number AY686687.1) was propagated in IBRS-2 cells using standard virology techniques and the supernatants of infected cells were clarified and stored at −80°C. Human embryonic kidney cells (HEK-293T, ATCC number CRL-11268) were maintained in Dulbecco’s Modified Eagle Media (DMEM, Invitrogen, USA) supplemented with 10% heat-inactivated FBS, 100 U/mL penicillin, and 10 µg/ml streptomycin sulfate. Arsenite (Sigma-Aldrich, USA), an inducer of SGs formation, was used to stimulate cells. The broad caspase inhibitor zVAD-FMK and the proteasome inhibitor MG132 were obtained from Beyotime (China).

### Plasmids

Foot-and-mouth disease virus 3C^pro^ (wild type, H46Y, C163G, and H181Y) constructs and the luciferase reporter plasmid nuclear factor-κB (NF-κB)-Luc have been described previously (11). The bicistronic reporter plasmid TK-Renilla luciferase(Rluc)-IRES-Fluc is constructed according to previous study (12). cDNA encoding porcine G3BP1 was amplified by standard RT-PCR from total RNA extracted from porcine peripheral blood mononuclear cells and cloned into pCAGGS-Flag encoding a N-terminal Flag-tag. Mutagenesis of G3BP1 (S149A, Q280A, E284A, and E288A) was carried out by Overlap Extension PCR using specific mutagenic primers (Table 1). All constructs were validated by DNA sequencing.

**Table 1:** Primers used in this study.

Primer	Sequences(5′–3′)
G3BP1-F	TTTGAATTCATGGTTATGGAGAAGCCTAGTCCCC
G3BP1-R	TTTAGATCTCACTGCCTTGGAGTAAGGCCCCTT
G3BP1-S149A-F	TCACGGAGCCTCAGGAGGAAGCCGAAGAAG
G3BP1-S149A-R	TTCCTCTACTTCTTCTTCGGCTTCCTC
G3BP1-Q280A-F	GTGCCAGCTTCAGCCCCTCGTCCAGAGACTAAACC
G3BP1-Q280A-R	TGGACGAGGGGCTGAAGCTGGCACTTTAACAACAT
G3BP1-E284A-F	CAGCCTCGTCCAGCCACTAAACCTGAATCTCAGAT
G3BP1-E284A-R	TTCAGGTTTAGTGGCTGGACGAGGCTGTGAAGCTG
G3BP1-E288A-F	GAGACTAAACCTGCCTCTCAGATTCCACCGCAGAG
G3BP1-E288A-R	TGGAATCTGAGAGGCAGGTTTAGTCTCTGGACGAG
G3BP1-1~284-R	GAAGATCTCACTCTGGACGAGGCTGTGAAGCTG
G3BP1-285~465-F	CCGGAATTCCGGATGACTAAACCTGAATCTCAGAT
qpG3BP1-F	CAAGAACCTGTATCTGAAGTC
qpG3BP1-R	TACTGTCTGTGCTATGTCTG
qFMDV-F	AACTCGTGCAACTTGGAACC
qFMDV-R	CATGCTCCGCTACGAAACAA
qpIFN-β-F	GCTAACAAGTGCATCCTCCAAA
qpIFN-β-R	AGCACATCATAGCTCATGGAAAGA
qpIP-10-F	ATGGTTCATCATCCCGAGCT
qpIP-10-R	CCAGGACTTGGCACATTCACT
qpRANTES-F	ACACCCTGCTGTTTTTCCTAC
qpRANTES-R	AGACGACTGCTGCCATGGA
qpIL-6-F	CTGCTTCTGGTGATGGCTACTG
qpIL-6-R	GGCATCACCTTTGGCATCTT
qpGAPDH-F	ACATGGCCTCCAAGGAGTAAGA
qpGAPDH-R	GATCGAGTTGGGGCTGTGACT

### Luciferase Reporter Gene Assays

Human embryonic kidney cells-293T cells or IBRS-2 cells grown in 48-well plate were co-transfected with a reporter plasmid and the indicated expression plasmids. Where indicated, cells were further infected with FMDV 30 h after the initial co-transfection. Cells were lysed 6 h later and firefly luciferase (Fluc) and Rluc activities were determined using the dual-luciferase reporter assay system (Promega, USA) according to the manufacturer’s protocol. The data represent relative Fluc activity normalized to Rluc activity and are representative of three independently conducted experiments. Data are presented as mean ± SD. Statistical values of *p < 0.05 were considered significant, and **p < 0.01 were considered highly statistically significant.

### Western Blotting and Phos-Tag Western Blotting Analyses

Briefly, HEK-293T cells or IBRS-2 cells cultured in 60-mm dishes were transfected with the indicated plasmids. Where indicated, IBRS-2 cells were further infected with FMDV 30 h after the initial co-transfection. After 30 h, the cells were harvested 6 h later by adding lysis buffer [25 mM Tris–HCl (pH 7.5), 150 mM NaCl, 1% Triton X-100, 20 nM phenylmethylsulfonyl fluoride], and protein concentrations were measured in whole cellular extracts. Equal amounts of the samples were then subjected to SDS-PAGE and analyzed for Flag-G3BP1 conjugated protein or endogenous G3BP1 protein by western blotting using an anti-Flag antibody (Macgene, China) or rabbit anti-G3BP1 (Abclonal, China), respectively. To confirm the expression levels of HA-tagged WT and mutant FMDV 3C^pro^, an anti-HA antibody (MBL, Japan) was used for immunoblotting. Expression of β-actin was detected with an anti-β-actin mouse mAb (Beyotime, China) to confirm loading of equal protein amounts. For phospho-G3BP1 studies, phosphorylation of G3BP1 at Ser-149 was verified by rabbit anti-phospho-G3BP1-Ser149 (Sigma). To confirm the expression levels of GAPDH, ubiquitin conjugating enzyme E2 I (UBE2I), ubiquitin conjugating enzyme E2 L3 (UBE2L3), ribosomal protein L15 (RPL15), chromosome 5 open reading frame 24 (C5ORF24) and FOS-like 2 (FOSL2), anti-GAPDH antibody (Beyotime, China), anti-UBE2I antibody (Proteintech, China), anti-UBE2L3 antibody (Proteintech, China), anti-RPL15 antibody (Proteintech, China), anti-C5ORF24 antibody (Proteintech, China), and anti-FOSL2 antibody (Proteintech, China) were used for immunoblotting. For phospho-RPL15, phospho-C5ORF24, and phospho-FOSL2 studies, the samples were subjected to Mn^2+^–Phos-tag SDS-PAGE using a 12% (w/v) polyacrylamide gel containing 50 µM of polyacrylamide-bound Mn^2+^–Phos-tag (Phos-tag AAL-107).

### Indirect Immunofluorescence Assay

Briefly, HEK-293T cells or IBRS-2 cells were seeded onto microscope coverslips, placed into 24-well dish, and allowed to reach approximately 80% confluence. After the indicated infection, transfection, or treatment, the cells were fixed with 4% paraformaldehyde for 15 min and then permeabilized with methyl alcohol for 10 min at room temperature. After three washes with TBST, the cells were blocked with TBST containing 5% bovine serum albumin for 1 h and then incubated separately with rabbit anti-G3BP1 antibody, rabbit anti-FMDV VP3 antibody, mouse anti-puromycin antibody, mouse anti-double-stranded RNA (dsRNA) antibody, mouse anti-Flag antibody, or mouse anti-HA antibody for 1 h. The cells were then treated with Alexa Fluor 488-labeled anti-mouse secondary antibody or Alexa Fluor 594-labeled anti-rabbit secondary antibody for 1 h at room temperature and subsequently treated with 4′,6-diamidino-2-phenylindole (DAPI) for 15 min at room temperature. Fluorescent images were visualized and examined by using a confocal laser scanning microscope (Fluoview ver. 3.1; Olympus, Japan).

## Results

### Identification of Phosphoproteins in FMDV-Infected IBRS-2 Cells

**Figure 1:**
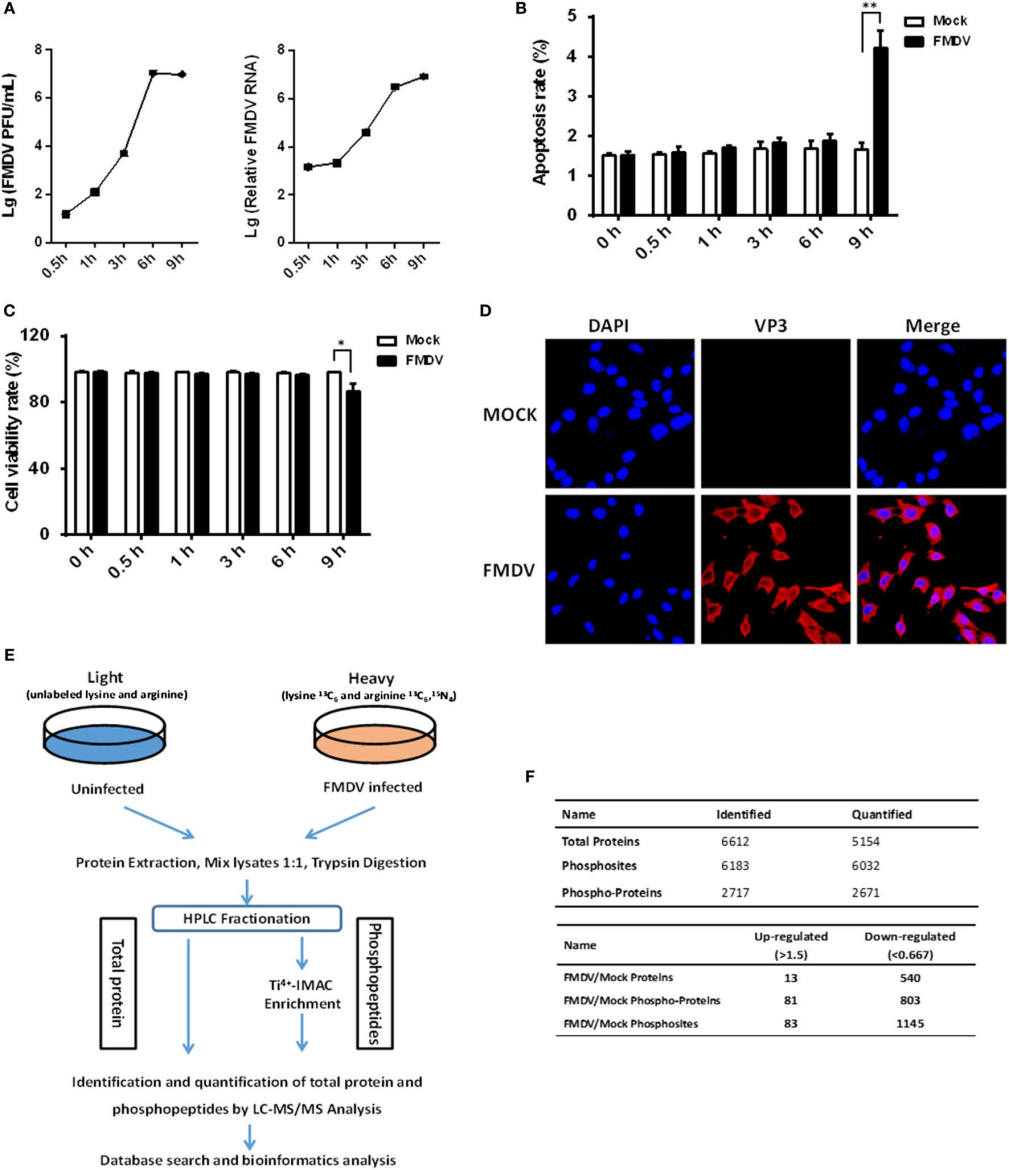
Experimental strategy of proteome and phosphoproteome analysis for foot-and-mouth disease virus (FMDV)-infected IBRS-2 cells. **(A)** IBRS-2 cells were infected with FMDV (MOI = 5) for the times indicated. After this, total cell lysates were prepared and analyzed for virus titer and genome RNA by virus plaque and real-time PCR. **(B,C)** The experiments were similarly performed as in **(A)**. Apoptosis and cell death by necrosis is analyzed by annexin V-FITC/PI apoptosis detection kit **(B)** and cytotoxicity lactate dehydrogenase activity assay kit **(C)**, respectively. **(D)** Dynamics of FMDV proliferation by immunofluorescence staining in the infected IBRS-2 cells at 6 h post-infection and mock-infected cells at 6 h as a control. **(E)** The workflow for the proteomics and phosphoproteomics experiments. **(F)** Total number of proteins, phosphoproteins, and phosphosites identification results when pooling the identifications of both biological replicates.

In order to get a comprehensive assessment of changes in host protein phosphorylation and signaling pathways induced by FMDV infection, we subjected control and FMDV-infected samples to SILAC coupled with phosphopeptide enrichment by Ti^4+^-IMAC affinity chromatography and tandem mass spectrometry (MS/MS)-based phosphoproteome analysis, combined with bioinformatics and functional assays. Foremost, the kinetics of FMDV RNA replication and host response of porcine kidney cells (IBRS-2) infected with FMDV (MOI = 5) were investigated. Viral RNA replication started at 1 hpi and reached peak levels at 6 hpi, as did progeny virus production (Figure 1A). At 9 hpi, significant cytopathic effects, apoptosis, and cell death by necrosis (as determined by lactate dehydrogenase activity assay) were observed (Figures 1B,C). The specific immunofluorescence corresponding to capsid protein VP3 of FMDV was readily detected in almost all of IBRS-2 cells infected with FMDV (MOI = 5) at 6 hpi, but no fluorescence was detected in mock-infected cells (Figure 1D). Based on this result, we chose 6 hpi for high-resolution, MS-based phosphoproteomics analyses to minimize any effects secondary to cell death/toxicity. Both FMDV-infected cells and control were lysed and followed by the digestion of protein samples all the way to peptide fractionation and phosphopeptide enrichment, and subsequently, liquid chromatography technique coupled with MS/MS (LC-MS/MS) analysis of the peptides (Figure 1E).

Two independent biological replicates were examined, which led to identification of a total of 6,032 phosphosites derived from 2,671 different phosphoproteins, with a false discovery rate of 1% at both the peptide and protein level (Figure 1F; Table S1 in Supplementary Material). Approximately 61.3% of all the phosphosites in FMDV-infected and control samples were identified in both replicates (Figure S1A in Supplementary Material), which is equivalent to phosphoproteomic datasets published previously (13). In both replicates, we also observed that phosphopeptides distributed evenly across the 7 HPLC fractions (Figure S1B in Supplementary Material). The resolution was excellent, with the vast majority of phosphopeptides (84%) eluting in only one or two fractions (Figure S1C in Supplementary Material), and the majority of phosphopeptides identified were singly phosphorylated (Figure S1D in Supplementary Material). Of the 2,671 phosphoproteins, 81 comprising 83 phosphosites were significantly upregulated (fold change >1.5, *p* < 0.05), and 803 comprising 1,145 phosphosites were significantly downregulated (fold change <0.67, *p* < 0.05) (Figure 1F). In cellular signaling, kinases are vital regulators for phosphorylation dynamics and in turn are frequently regulated by phosphorylation (14). Therefore, we examined the data for kinases that might play a key role in mediating a large number of downregulated phosphopeptides seen in the host phosphoproteome during FMDV infection. Interestingly, a total of 113 kinases were identified and quantified in the phosphoproteome analysis. Our research identified 40 kinases with downregulated phosphosites, while only one kinase, tyrosine-protein kinase ABL1, with upregulated phosphosites following FMDV infection (Table S2 in Supplementary Material). To further test whether changes in phosphorylation stemmed from changes in protein expression level, we also measured alterations of protein abundance in parallel. Of a total of 884 regulated phosphoproteins, only 5.9% (53 proteins) could be attributed to altered protein abundance (Tables S3 and S4 in Supplementary Material). Therefore, the alteration of phosphosite levels was principally driven by kinase and phosphatase activities in response to FMDV infection.

### Validation of Protein and Phosphoprotein Identification by Western Blotting and Phos-Tag Western Blotting

**Figure 2:**
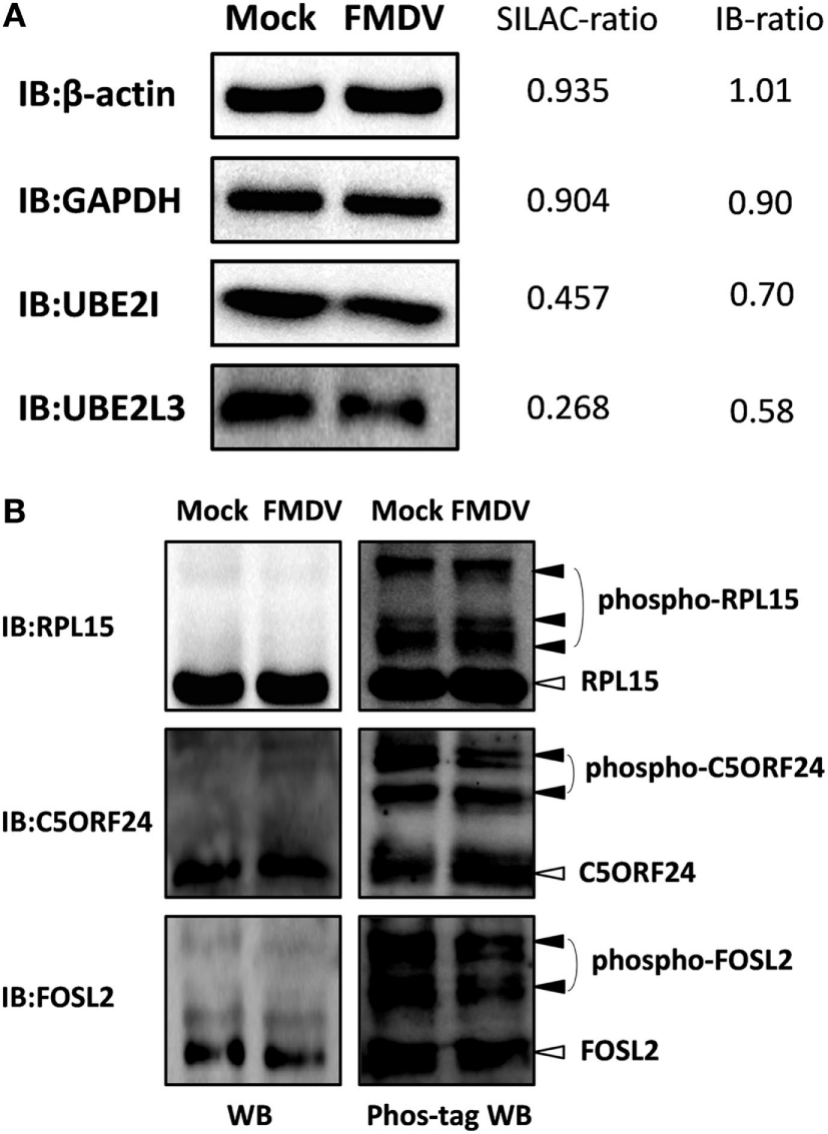
Confirmation of differentially expressed proteins and phosphoproteins by western blotting and Phos-tag western Blotting. **(A)** Analysis of ubiquitin conjugating enzyme E2 I, ubiquitin conjugating enzyme E2 L3, glyceraldehyde-3-phosphate dehydrogenase, β-actin expression levels in foot-and-mouth disease virus (FMDV)-infected and control cells by western blotting. SILAC-ratios and immunoblotting ratios (infection/control) were shown on the right side. **(B)** Analysis of the dynamic phosphorylation alterations of the three differentially phosphoproteins (ribosomal protein L15, chromosome 5 open reading frame 24, and FOS-like 2) in FMDV-infected and control cells by Phos-tag western blotting.

To confirm the LC−MS/MS data, four representative proteins (β-actin, GAPDH, UBE2I, and UBE2L3) were selected for immunoblotting analysis with antibodies specific for these proteins. Western blotting results confirmed the downregulation of UBE2I and UBE2L3, and the unchange of β-actin and GAPDH between FMDV-infected and uninfected cells (Figure 2A). SILAC-ratios and immunoblotting ratios (infection/control) were shown on the right side (Figure 2A).

Because almost no phosphospecific antibodies against porcine protein are available, Phos-tag western blotting has been developed to detect protein phosphorylation and we select three representative proteins (RPL15, C5ORF24, and FOSL2) to confirm the phosphoproteomic data. We first performed normal SDS-PAGE analysis using a 12% (w/v) polyacrylamide gel. Successive immunoblotting with the anti-RPL15, anti-C5ORF24, or anti-FOSL2 antibody showed that RPL15, C5ORF24, and FOSL2 appeared as a single band, not showing any mobility shifts of phosphorylated proteins. Next, we subjected the same samples to Mn^2+^–Phos-tag SDS-PAGE using a 12% (w/v) polyacrylamide gel containing 50 µM of polyacrylamide-bound Mn^2+^–Phos-tag. Regarding immunoblotting analysis with the anti-RPL15, anti-C5ORF24, and anti-FOSL2 antibodies, several additional upshifted bands were observed compared with those in regular western blotting. The results presented a difference in the mobility between the phosphorylated proteins and the corresponding dephosphorylated proteins. Together, the Phos-tag western blotting results showed that the phosphorylation levels of C5ORF24 and FOSL2 were significantly decreased while that of RPL15 remained unchanged in FMDV-infected cells (Figure 2B), in agreement with the phosphoproteomic data (Table S4 in Supplementary Material).

### FMDV Infection Shuts Off Host Cap-Dependent Translation, but Not Cap-Independent Translation for Viral Proteins

**Figure 3:**
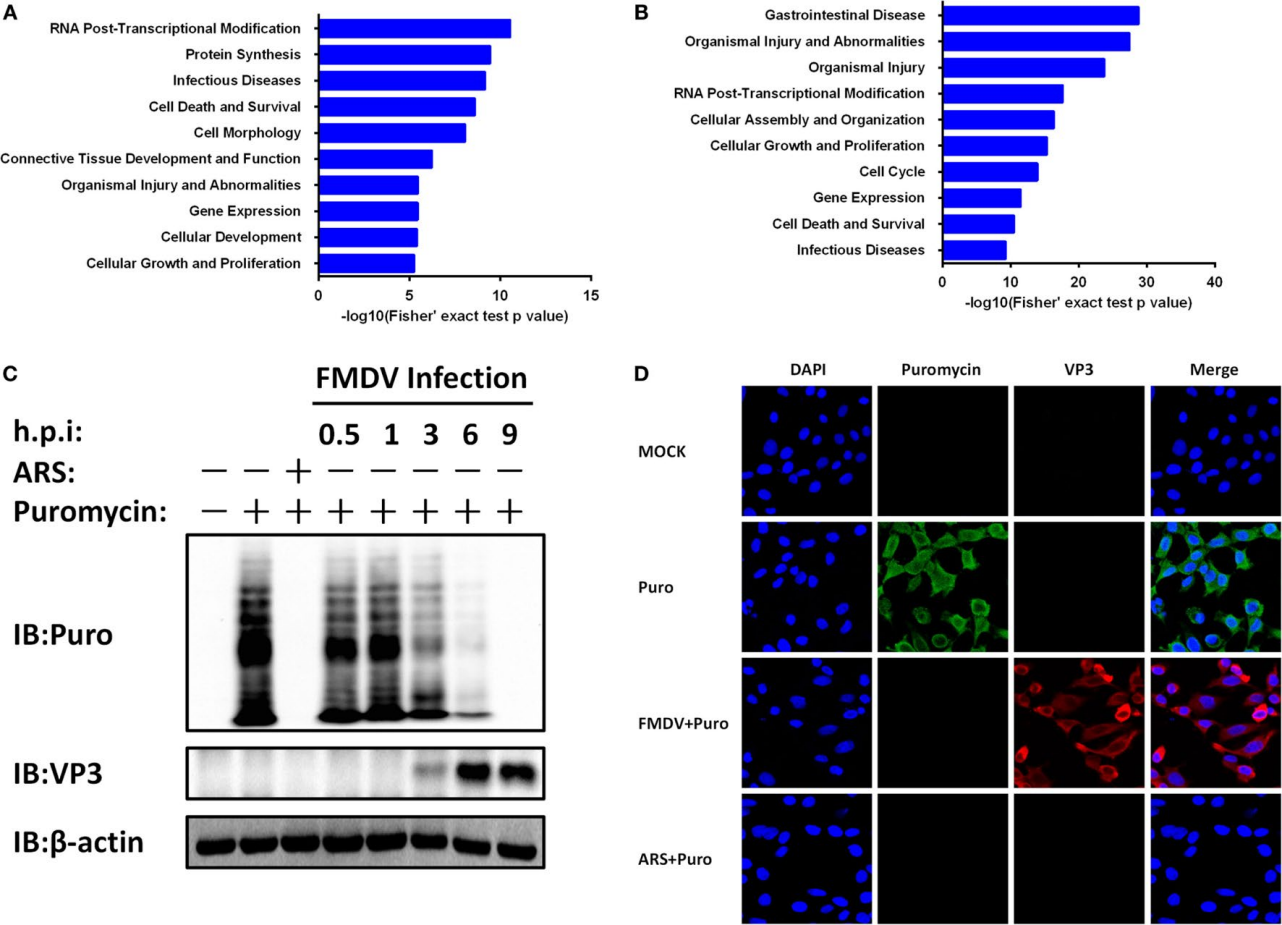
Foot-and-mouth disease virus (FMDV) infection-induced host cap-dependent translation but not cap-independent translation shutoff. **(A,B)** Biological function categories analysis by Ingenuity pathway analysis (IPA^®^). The top 10 biological function categories relevant to the regulated proteome **(A)** and phosphoproteome **(B)** are shown with the corresponding score (−log [*p* value]). **(C)** IBRS-2 cells were mock-infected or infected with FMDV at 5 MOI for 0.5, 1, 3, 6, and 9 h, or treated with arsenite for 30 min at 37°C. Puromycin (25 µg/ml) was then added to the medium in each well for 15 min. The cells were lysed and analyzed by western blotting. Protein synthesis was determined using puromycin labeling followed by immunoblot with the anti-puromycin mAb. FMDV VP3 level was monitored by immunoblot with anti-FMDV VP3 protein antibodies. Beta-actin (β-actin) detection was used to confirm equal sample loading. **(D)** IBRS-2 cells were mock-infected or infected with FMDV at 5 MOI for 6 h, or treated with arsenite for 30 min at 37°C. Puromycin (25 µg/ml) was then added to the medium in each well for 15 min. The cells were fixed for indirect immunofluorescence assays with the following antibodies: rabbit anti-FMDV VP3 (red) and mouse anti-puromycin (green). Nuclei were counterstained with DAPI (blue). Fluorescent images were acquired with a confocal laser scanning microscope.

To gain biological insight into the proteome and phosphoproteome data, we performed in-depth bioinformatics characterization of the datasets. As the first step, 553 regulated proteins (Figure 1F; Table S3 in Supplementary Material) and 884 regulated phosphoproteins (Figure 1F; Table S1 in Supplementary Material) upon FMDV infection were analyzed for biological function categories using the Ingenuity Pathway Analysis (IPA^®^). The top 10 ranked biological function categories for both proteome and phosphoproteome datasets included RNA post-transcriptional modification, gene expression, and infectious diseases (Figures 3A,B). Within these top categories we identified a large number of proteins known as RNA-binding proteins (RBPs), which are involved in gene expression, RNA processing, and RNA splicing (15). Previous studies have shown that RBPs play key roles in post-transcriptional control of RNAs, which, along with transcriptional regulation, are responsible for regulating the patterns of gene expression during virus infection (3). The modification of the host translation machinery is subverted to favor viral RNA translation and replication. The mechanisms by which picornavirus infections achieve inhibition of cellular mRNAs translation reside mainly in the cleavage of host RBPs [such as eukaryotic translation initiation factor (eIF) 4 G (eIF4G), eIF4AI, eIF3, eIF5B, and poly(A) binding protein] by viral proteases L^pro^ or 3C^pro^, as well as in the phosphorylation of eIF2α and eIF4E-BP (5, 6, 16–19). Here, our proteome data showed that eIF4G, eIF3, and eIF2α were downregulated (Table S3 in Supplementary Material), and eIF4E-BP was dephosphorylated following FMDV infection in one of the biological replicates (Table S1 in Supplementary Material). IPA further showed that all of these RBPs are associated with host cap-dependent translation initiation, suggesting that FMDV infection results in inhibition of host protein synthesis and, in general, shutdown of cellular cap-dependent gene expression (Figure S2A in Supplementary Material).

To test whether protein synthesis inhibition is due to FMDV infection, we infected IBRS-2 cells with FMDV for different times and protein synthesis was determined using puromycin labeling followed by immunoblot with the anti-puromycin mAb. Puromycin blocks translation by entering the A-site of ribosomes and is itself transferred to the growing peptide chain, resulting in the disassembly of polysomes and release of truncated nascent polypeptides, which will contain puromycin instead of normal amino acid at their C-terminus. Compared with uninfected cells, host protein levels decreased a time-dependent manner (Figure 3C).

A subset of RNA viruses can initiate cap-independent translation of their mRNAs *via* IRES structures, thereby maintaining translation when cap-dependent translation is compromised (3). To determine whether the expression of FMDV proteins is affected by cap-dependent translation shutoff, we infected IBRS-2 cells with FMDV for different times and analyzed viral capsid protein VP3 levels by western blotting. Compared with uninfected cells, FMDV VP3 protein levels increased a time-dependent manner (Figure 3C). Even with appreciable cytopathic effects, apoptosis and cell death by necrosis, VP3 protein levels continued to rise and peaked at 9 hpi. In addition, the proteome and phosphoproteome data also identified eight FMDV mature proteins (L^pro^, VP2, VP3, VP4, 2A, 2B, 2C, and 3C^pro^) exclusively in the infected samples (Table S3 in Supplementary Material). In addition, four viral phosphopeptides, derived from the 2B and 3D proteins (Table S1 in Supplementary Material), were identified with high confidence.

To further confirm these results above, IBRS-2 cells were mock-infected or infected with FMDV at 5 MOI for 6 h, or treated with arsenite for 30 min at 37°C. Puromycin (25 µg/ml) was then added to the medium in each well, and after 15 min the cells were washed for three times with PBS, fixed, permeabilized, and processed for IFA. In mock-infected cells, strong cytoplasmic puromycin staining was detected while in virus-infected cells specific fluorescence corresponding to capsid protein VP3 of FMDV was detected (Figure 3D). Very little puromycin was detected in arsenite-treated and FMDV-infected cells (Figure 3D). Together, these data suggest that FMDV infection shuts off host cap-dependent translation, but not cap-independent translation of viral proteins.

### Dephosphorylation of the G3BP SG Assembly Factor 1 Is Involved in Maintaining FMDV IRES Activity

**Figure 4:**
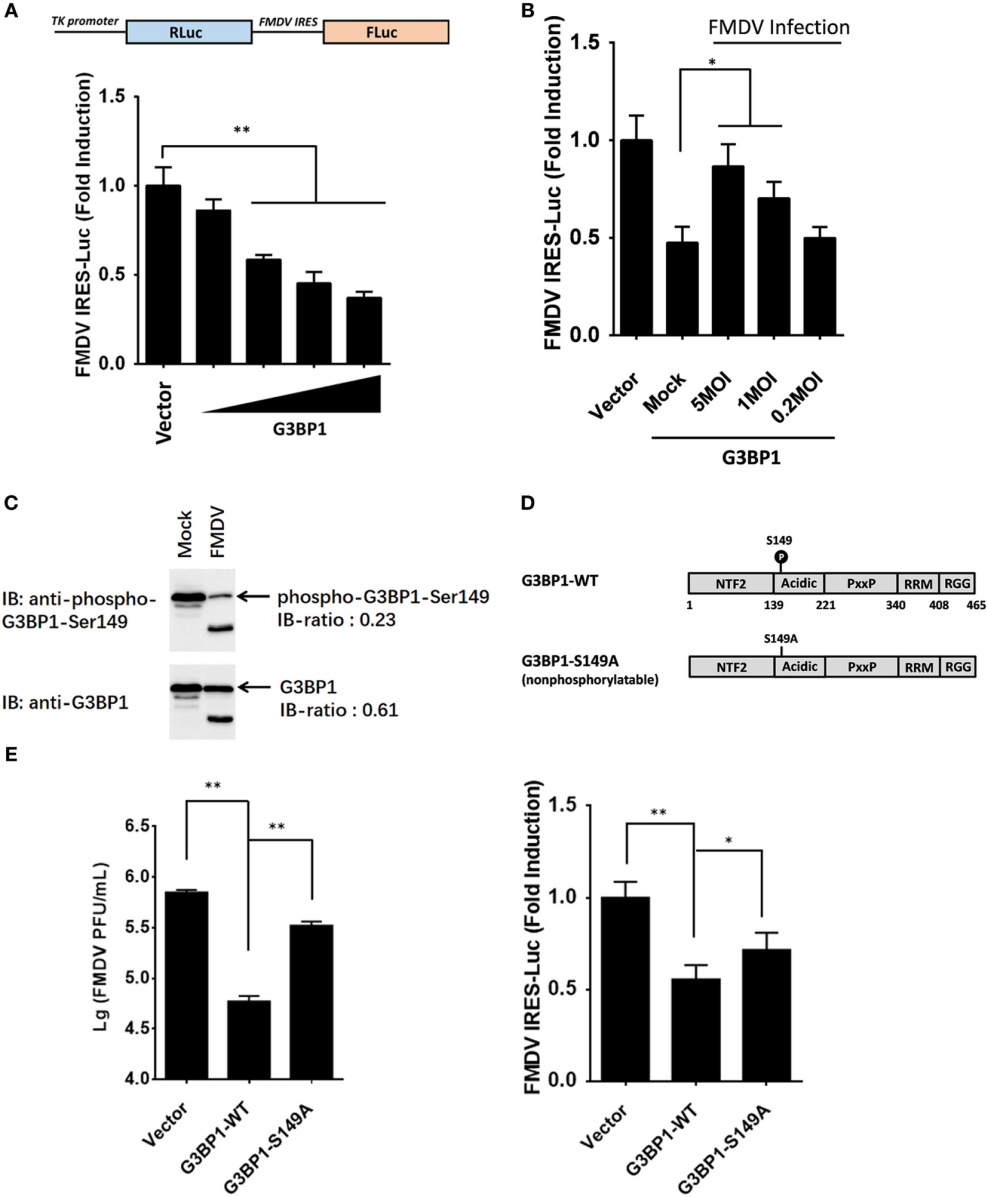
Dephosphorylation of G3BP1 is involved in foot-and-mouth disease virus (FMDV) internal ribosome entry site (IRES) activity. **(A)** Schematic representation of the construct TK-Renilla luciferase(Rluc)-IRES-Firefly luciferase(Fluc). IBRS-2 cells were transfected with TK-Rluc-IRES-Fluc along with increasing quantities (0, 0.125, 0.25, or 0.5 µg) of plasmid encoding porcine G3BP1, using Lipofectamine 2000. Luciferase assays were performed 36 h after transfection. **(B)** IBRS-2 cells were transfected with TK-Rluc-IRES-Fluc along with porcine G3BP1 expression plasmid or an empty vector (0.5 µg). 30 h after initial transfection, cells were infected with FMDV at different MOIs. Luciferase assays were performed 6 h after FMDV infection. **(C)** IBRS-2 cells were infected with FMDV (MOI = 5). After 6 h post-infection, total cell lysates were prepared and analyzed with western blotting with rabbit anti-G3BP1 and rabbit anti-phospho-G3BP1-Ser149 antibodies. Immunoblotting ratios (infection/control) were quantified by ImageJ. **(D)** Schematic representation of wild-type porcine G3BP1 and its derivatives. The domains of porcine G3BP1 were annotated analogously to those of human G3BP1. IBRS-2 cells were transfected with Flag-tagged wild-type G3BP1 or its mutants as indicated, along with TK-Rluc-IRES-Fluc. Luciferase assays were performed at 36 h after the transfection. **(E)** IBRS-2 cells were transfected with the designated flag-tagged porcine G3BP1 expression plasmids or its mutants or an empty vector, and, 30 h later, the cells were infected with FMDV (MOI = 5). 6 h after FMDV infection, virus plaque was performed to analyze for virus titer.

The IRES of FMDV is one of the strongest IRES described to date (2). However, given the large complexity of FMDV IRES, the mechanistic basis for its mode of action is not fully understood. With many in depth RNA–protein interaction studies having been performed with FMDV IRES, the list of IRES-transacting factors (ITAFs) is growing incessantly (20–28). We examined the proteome and phosphoproteome data for identified ITAFs that might play a key role in maintaining FMDV IRES activity (Table S5 in Supplementary Material). Considering that most of these ITAFs have been investigated for their roles in IRES activity, such as eIF4G, eIF4A, Gemin5, PCBP2 [poly(rC) binding protein 2], PTBP1 (polypyrimidine tract binding protein 1), and hnRNPK (heterogeneous nuclear ribonucleoprotein K) (21–29), we focused our attention on the RBPs not well studied previously. Riboproteomic analysis of proteins interacting with the IRES of FMDV viral RNA has shown that G3BP1 interacts with FMDV IRES elements (20). IPA analysis of G3BP1 network also suggested that this protein interact with many other RBPs, and be involved in mRNA localization, mRNA translation, and virus replication (Figures S2B,C in Supplementary Material). However, little is known about the effect of G3BP1 on FMDV IRES. In subsequent experiments, we focused on this novel ITAF of picornavirus IRES, and examined whether G3BP1 regulates FMDV IRES activity. To gauge the effect on FMDV IRES activity, we utilized a bi-cistronic reporter construct, TK-Rluc-IRES-Fluc, that allows the expression of Fluc under control of FMDV IRES, and Rluc expression directed by cap-dependent translation initiation (Figure 4A). Overexpression of G3BP1 significantly inhibited FMDV IRES activity, compared with that of an empty plasmid control (Figure 4A). However, the inhibition on FMDV IRES by G3BP1 was alleviated by FMDV infection in a dose-dependent manner (Figure 4B).

Consistent with previous work (9), three phosphosites of G3BP1 are identified by phosphoproteome analysis (Table S1 in Supplementary Material). The phosphorylation at Ser-149 was downregulated in FMDV-infected cells (Figure 4C), whereas phosphorylations at Ser-231 and Ser-252 had no significant changes (Table S1 in Supplementary Material), compared with uninfected cells. To determine whether phosphorylation at Ser-149 affected the ability of G3BP1 to inhibit IRES, a non-phosphorylatable G3BP1 mutant (S149A) was constructed. The S149A mutant impaired the ability of G3BP1 to inhibit IRES (Figure 4D). Thus, dephosphorylation of G3BP1 at Ser-149 residue in FMDV-infected cells (Figure 4C) may contribute to the FMDV reversal of G3BP1-mediated inhibition on viral IRES (Figure 4D). To further determine the impact of FMDV-induced G3BP1 dephosphorylation on FMDV replication, we transfected cells with the WT G3BP1 expression plasmids or its mutant as indicated for 24 h, and then challenged cells with FMDV. Overexpression of wild-type porcine G3BP1 significantly inhibited FMDV replication as revealed by progeny virus production (Figure 4E). Interestingly, the non-phosphorylatable G3BP1 mutant (S149A) impaired the antiviral activity of G3BP1 (Figure 4E). These data confirm that G3BP1 is a host factor that suppresses FMDV replication and that its antiviral activity depends on its phosphorylation.

### FMDV Disrupts G3BP1-Induced SG Assembly by Cleaving G3BP1

**Figure 5:**
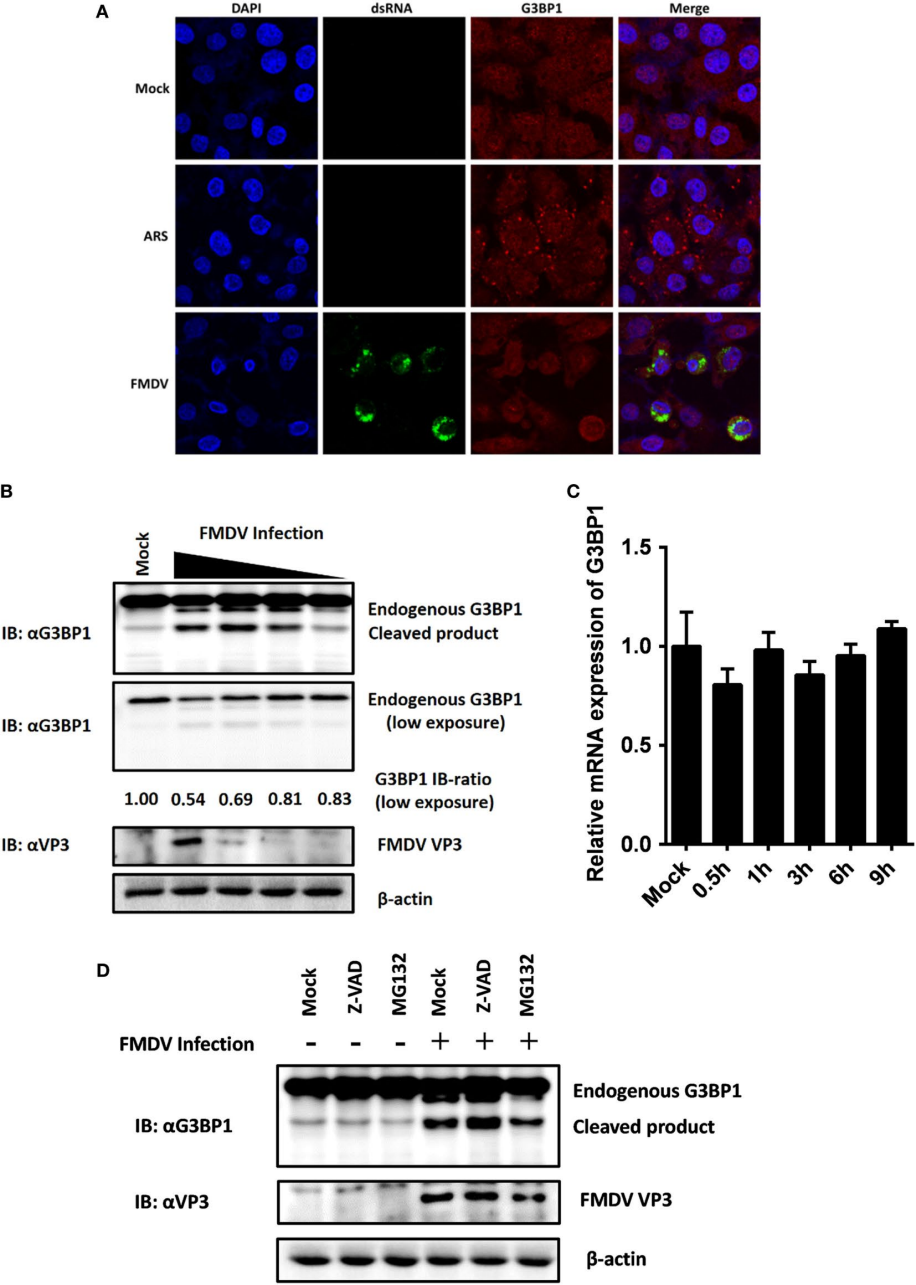
Foot-and-mouth disease virus (FMDV) infection induces G3BP1 cleavage. **(A)** IBRS-2 cells were mock-infected or infected with FMDV (MOI = 5). At 6 h post-infection (hpi), indirect immunofluorescence assays were performed. Arsenite, an inducer of stress granules formation, was used to stimulate cells for 1 h. The cells were fixed and incubated with a monoclonal antibody against virus double-stranded RNA (green) and a polyclonal antibody against G3BP1 (red). The nuclei of cells were stained with DAPI (blue). **(B)** IBRS-2 cells were mock-infected or infected with FMDV at different MOIs (0.04, 0.2, 1, and 5 MOI), lysed at 6 hpi, and analyzed for G3BP1 protein levels by western blotting. Immunoblotting ratios (infection/control) were quantified by ImageJ. **(C)** IBRS-2 cells were mock-infected or infected with FMDV for different times (0.5, 1, 3, 6, and 9 h), lysed and analyzed for G3BP1 mRNA levels by real-time reverse transcriptase-PCR. **(D)** IBRS-2 cells were mock-infected or infected with FMDV (MOI = 5). MG132 or zVAD-FMK was added to the indicated group at a final concentration of 20 µM. Cell lysates were prepared 6 h after infection/treatment and analyzed by western blotting.

Dephosphorylation of G3BP1 at Ser-149 has been shown to induce SGs assembly following arsenite treatment (30). SGs are protein-mRNA aggregates formed in response to environmental stresses, resulting in translational inhibition (31). Since G3BP1 is a key component and commonly used marker of SGs (32), we investigated the status of G3BP1-induced SGs during FMDV infection. IBRS-2 cells were infected for 6 h and then immunostained with antibody against dsRNA, a viral RNA replicative intermediate, and with antibody against G3BP1, to determine whether FMDV-infected cells contained SGs. We observed that, despite efficient G3BP1 dephosphorylation (Figure 4C), FMDV infection failed to induce SG accumulation, compared with arsenite-treated cells (Figure 5A).

The absence of SGs following dephosphorylation of G3BP1 at Ser-149 suggested that FMDV infection might weaken the ability of cells to assemble SGs. Previous studies have shown that SG inhibition could be driven by the interaction, sequestration, or cleavage of the SGs marker G3BP1 (33–39). As shown in Figure 4C, in addition to inducing dephosphorylation of full-length G3BP1, FMDV infection decreased G3BP1 protein abundance, concomitant with the appearance of a faster migrating protein band, presumably a cleavage product of G3BP1. To further determine the nature of this G3BP1 antibody-reacting product, IBRS-2 cells were mock-infected or infected with FMDV at increasing MOIs (0.04, 0.2, 1, and 5 MOI) for 6 h, and lysates were prepared, fractionated on SDS-PAGE, and analyzed by immunoblotting. Compared with uninfected cells, G3BP1 protein abundance was substantially reduced in FMDV-infected IBRS-2 cells in a dose-dependent manner. Again, this was coincided with the appearance of a faster migrating band, more obvious in cells infected at higher MOIs (Figure 5B). The levels of G3BP1 mRNA were comparable between mock-infected and FMDV-infected cells, regardless of post-infection times (Figure 5C), suggesting that the reduction of G3BP1 protein abundance is not due to decrease in G3BP1 mRNA expression. A previous study showed that melanoma differentiation-associated gene 5, a cytoplasmic viral innate immune sensor, is cleaved in poliovirus-infected cells by cellular caspases as well as the host proteasome (40). We considered such a possibility for FMDV-induced cleavage of G3BP1. However, neither the broad caspase inhibitor zVAD-FMK nor the proteasome inhibitor MG132 abrogated the ability of FMDV to induce G3BP1 cleavage (Figure 5D), indicating that the cleavage of G3BP1 observed in FMDV-infected cells was most probably independent of the apoptosis and proteasome pathways.

### FMDV 3C Proteinase Proteolytically Cleaves G3BP1

**Figure 6:**
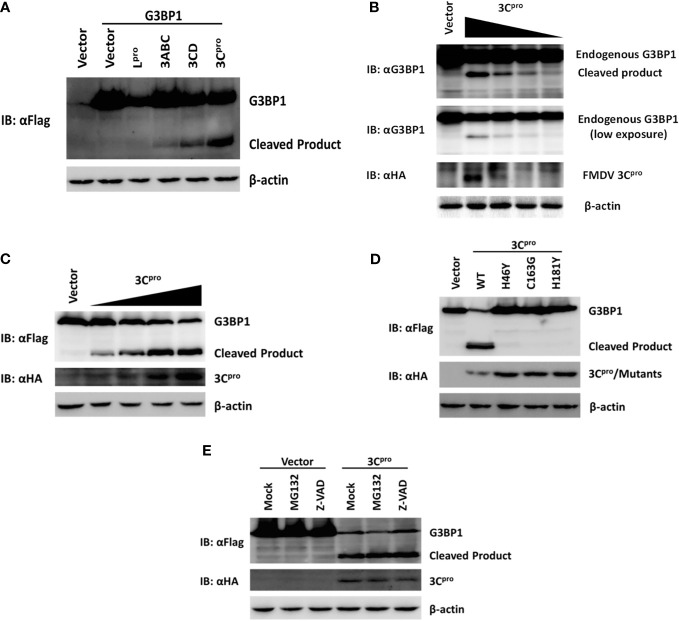
Foot-and-mouth disease virus 3C^pro^ cleaves G3BP1 by means of its protease activity. **(A)** Human embryonic kidney cells (HEK)-293T cells cultured in 60-mm dishes were transfected with Flag-tagged porcine G3BP1 as indicated (4 µg), along with HA-L^pro^, 3C^pro^, or 3C^pro^-containing precursors (0.05 µg). Cell lysates were prepared 30 h post-transfection and analyzed by western blotting. **(B)** IBRS-2 cells were transfected with increasing quantities (0, 0.5, 1, 2, or 4 µg) of plasmid encoding 3C^pro^. Cell lysates were prepared 36 h post-transfection and analyzed by western blotting. **(C)** HEK-293T cells were transfected with Flag-tagged wild-type porcine G3BP1 (4 µg), along with increasing quantities HA-3C^pro^ plasmid (0, 0.0125, 0.025, 0.05, or 0.1 µg). Cell lysates were prepared 30 h post-transfection and analyzed by western blotting. **(D)** HEK-293T cells were transfected with Flag-tagged porcine G3BP1 expression plasmid (4 µg), along with wild-type 3C^pro^ expression plasmids or its mutants (0.05 µg). Cell lysates were prepared 30 h post-transfection and analyzed by western blotting. **(E)** HEK-293T cells were co-transfected with Flag-tagged porcine G3BP1 expression plasmid (4 µg) and plasmid encoding 3C^pro^ or empty vector (0.05 µg). 24 h after transfection, MG132 or zVAD-FMK were added to a final concentration of 20 µM. Cell lysates were prepared 8 h after treatment and analyzed by western blotting.

In addition to processing the viral polyprotein, FMDV 3C proteinase and leader proteinase can cleave cellular proteins such as eIF4A and eIF4G to regulate translation during infection (5, 6). Because we found FMDV-mediated G3BP1 cleavage was independent of the host apoptosis and proteasome pathways, we investigated whether an FMDV proteinase(s) is directly responsible. As shown in Figure 6A, overexpression of 3C^pro^ resulted in reduction in full-length G3BP1 protein abundance and appearance of a shorter product, resembling the pattern observed in FMDV-infected cells (Figure 5B). The processing intermediates encompassing 3C^pro^, i.e., 3ABC and 3CD, also processed G3BP1 with the same cleavage pattern, albeit less efficiently than mature 3C^pro^ (Figure 6A). Interestingly, overexpression of L^pro^ also reduced the protein abundance of G3BP1. Nevertheless, no cleavage products were observed, suggesting G3BP1 is unlikely a proteolytic substrate for L^pro^ (Figure 6A). We speculate it may result from inhibition of cellular translational machinery following L^pro^-mediated proteolysis of the translation initiation factor eIF4G. This notion will need to be validated in future studies. Importantly, both endogenous G3BP1 and the ectopically co-expressed, Flag-tagged porcine G3BP1 were cleaved by 3C^pro^, and the degree of cleavage was correlated with the expression level of 3C^pro^ (Figures 6B,C).

Foot-and-mouth disease virus 3C^pro^ is a cysteine proteinase responsible for most cleavages within the viral polyprotein, same as the 3C^pro^ of other picornaviruses (2). For FMDV 3C^pro^, residues His-46 and Cys-163 are part of the catalytic triad and His-181 is part of the binding pocket (41). Mutations at any of these residues [Tyr for His-46 (H46Y), Gly for Cys-163 (C163G), or Tyr for His-181 (H181Y)] abrogated the capacity of FMDV 3C^pro^ to proteolytically process viral P1, P2, or P3 substrates in *trans* (42). In order to determine whether 3C^pro^ cleaves G3BP1 by means of its protease activity, we compared the abilities of WT and various catalytically inactive mutants (H46Y, C163G, and H181Y) of 3C^pro^ to cleave ectopically co-expressed Flag-G3BP1. The result suggested that only WT 3C^pro^-induced G3BP1 cleavage, while none of the 3C^pro^ mutants was able to do so (Figure 6D). These data suggest that G3BP1 is a proteolytic substrate for 3C^pro^. We confirmed that the cleavage of G3BP1 observed in 3C^pro^-expressing cells was also independent of the apoptosis and proteasome pathways (Figure 6E).

### FMDV 3C^pro^ Cleaves G3BP1 at Glutamic Acid-284

**Figure 7:**
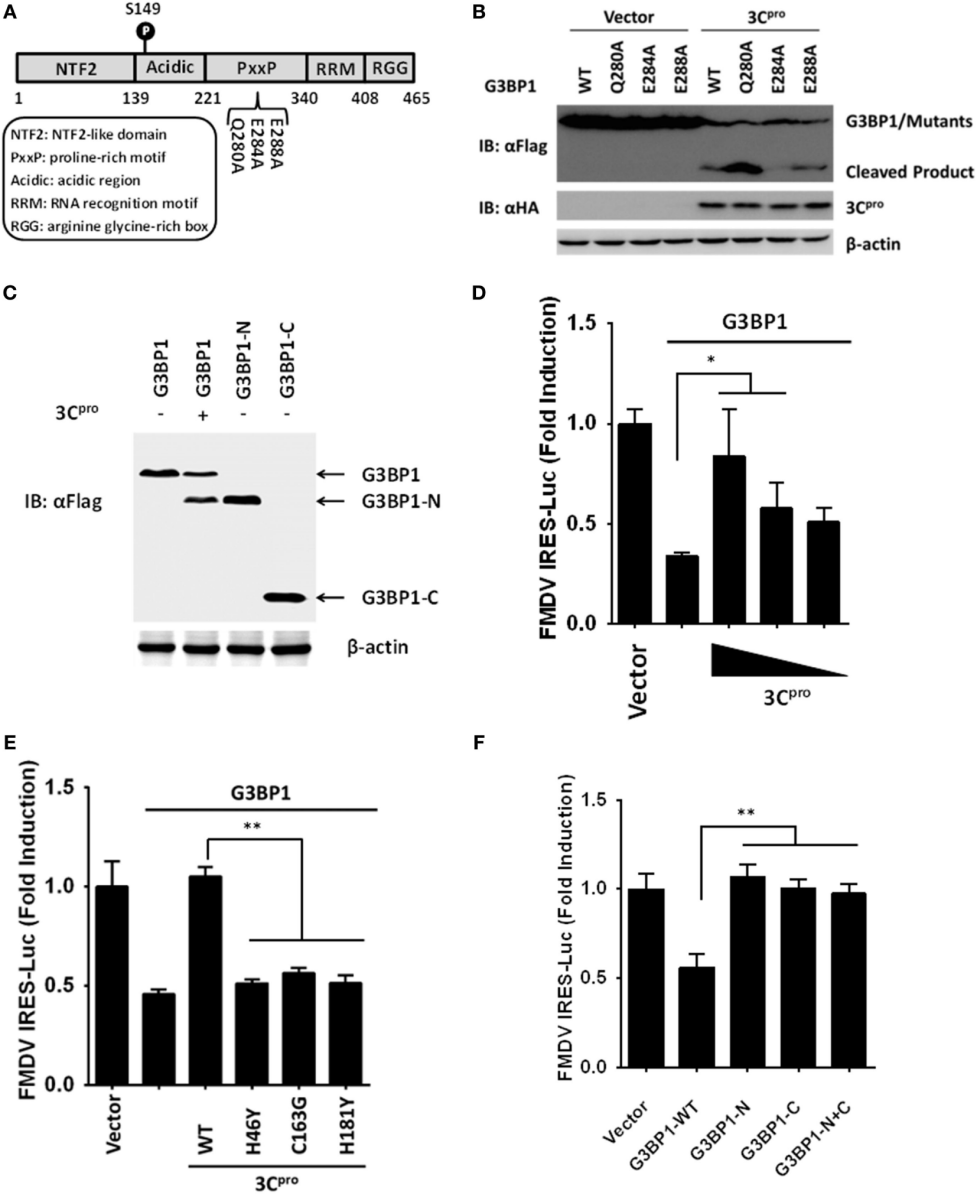
Foot-and-mouth disease virus 3C^pro^ cleaves G3BP1 at E284 and inactivates G3BP1-mediated inhibition of internal ribosome entry site (IRES) activity. **(A)** Schematic representation of wild-type porcine G3BP1 and its derivatives. **(B,C)** Human embryonic kidney cells-293T cells cultured in 60-mm dishes were transfected with Flag-tagged wild-type porcine G3BP1 or its mutants as indicated, along with HA-3C^pro^ or empty vector. Cell lysates were prepared 30 h post-transfection and analyzed by western blotting. **(D)** IBRS-2 cells were transfected with TK-Renilla luciferase(Rluc)-IRES-Firefly luciferase(Fluc) (0.05 µg), and Flag-tagged porcine G3BP1 expression plasmid (0.3 µg), along with increasing quantities of the plasmid encoding 3C^pro^ (0.025, 0.05, and 0.1 µg). Luciferase assays were performed at 36 h after the transfection. **(E)** IBRS-2 cells were transfected with TK-Rluc-IRES-Fluc (0.05 µg), and Flag-tagged porcine G3BP1 expression plasmid (0.3 µg), along with the designated 3C^pro^ expression plasmids (0.1 µg). Luciferase assays were performed at 36 h after the transfection. **(F)** IBRS-2 cells were cotransfected with TK-Rluc-IRES-Fluc, and either a plasmid encoding Flag-fused wild-type porcine G3BP1, a plasmid encoding putative 3C^pro^-induced cleavage fragments of G3BP1, or an empty vector (0.4 µg). Luciferase assays were performed at 36 h after the transfection.

We next examined for potential 3C^pro^ cleavage sites in the sequence of porcine G3BP1. Previous investigations on 3C^pro^ substrate specificity recognized a partiality at the P1 position for both glutamine (Gln, Q) and/or glutamic acid (Glu, E) (41). We focused on the sequence of G3BP1 for potential 3C^pro^ cleavage sites at which scissions could lead to fragments of the observed size. A series of G3BP1 mutants were constructed in which the invariant Gln or Glu at each potential P1 position was substituted with alanine (Ala, A), and examined their cleavage by 3C^pro^ (Figure 7A). The E284A mutation blocked the cleavage event, yielding only full-length G3BP1 protein in the presence of 3C^pro^ (Figure 7B). In contrast, Q280A and E288A mutations had no impact on 3C^pro^-mediated G3BP1 cleavage. To further confirm that G3BP1 is cleaved at E284 by FMDV 3C^pro^, a DNA vector encoding the putative N-terminal fragment of G3BP1 (residues 1–284) was transfected into cells. The Flag-tagged G3BP1 (1–284) fragment gave rise to a protein band having the same size as the putative cleavage product of N-terminally Flag-tagged G3BP1 by 3C^pro^ (detected by anti-Flag antibody) (Figure 7C). Taken collectively, these data suggest that glutamic acid-284 (E284) is the cleavage site at which 3C^pro^ scissors G3BP1.

### FMDV 3C Proteinase Negates G3BP1-Mediated Inhibition on FMDV IRES

To understand the biological significance of 3C^pro^-mediated G3BP1 cleavage in FMDV-infected cells, we asked the question as to whether 3C^pro^ alleviates the inhibitory effect of G3BP1 on FMDV IRES. Indeed, overexpression of 3C^pro^ dose-dependently rescued the FMDV IRES activity in the presence of G3BP1 overexpression (Figure 7D). In contrast, the catalytically inactive 3C^pro^ mutants were not effective (Figure 7E), consistent with their inability to cleave G3BP1 (Figure 6D). To further determine whether either of the 3C^pro^-induced cleavage fragments of G3BP1 retains the inhibitory potential on FMDV IRES, we ectopically expressed the N-terminal fragment of G3BP1 (residues 1–284), or the C-terminal G3BP1 fragment (residues 285–465) that would result from 3C^pro^ cleavage, and examined their effects on FMDV IRES. While full-length G3BP1 significantly downregulated IRES activity, neither N-terminal nor C-terminal fragment had such an effect (Figure 7F). We thus conclude that 3C^pro^-mediated cleavage of G3BP1 nullifies the ability of G3BP1 to suppress FMDV IRES-dependent translation of viral polyprotein.

### 3C^pro^-Induced G3BP1 Cleavage Fragments Lost the Ability to Induce SGs Assembly

As mentioned above, the finding that FMDV infection does not induce SGs raised the possibility that 3C^pro^-mediated G3BP1 cleavage could disrupt the assembly of SGs by disconnecting the cellular signals that mediate the accumulation of SGs. To determine whether 3C^pro^ blocks SG assembly in response to exogenous stress triggers, we monitored arsenite-induced SG formation in 3C^pro^-transfected cells. In control vector-transfected cells, arsenite-induced SGs were detected in almost every cell. In contrast, no significant induction of SG assembly was detected in FMDV 3C^pro^-expressing cells (Figure S3A in Supplementary Material). We next determined whether the absence of SGs formation was attributed to the protease activity of 3C^pro^. We found arsenite treatment efficiently induced SGs in cells overexpressing a catalytically inactive mutant (C163G) of 3C^pro^ (Figure S3A in Supplementary Material). This result is consistent with the inability of this 3C^pro^ mutant to cleave G3BP1.

Previous studies have suggested that the NTF2-like and RNA-binding domains mediate the G3BP1 recruitment to SGs (43). Because 3C^pro^ targeted G3BP1 at a specific residue (E284) between the NTF2-like and the RNA-binding domain, we next determined whether either of the 3C^pro^-generated cleavage fragments of G3BP1 is recruited to SGs. Cells were transfected with DNA constructs encoding wild-type porcine G3BP1 or either of the two putative fragments, followed by treatment with arsenite. Whereas wild-type porcine G3BP1 decorated the SGs, the N-terminal and C-terminal fragments did not (Figure S3B in Supplementary Material), suggesting that 3C^pro^-mediated cleavage of G3BP1 efficiently impaired SGs assembly.

### G3BP1 Negatively Regulates FMDV Replication by Inducing Antiviral Innate Immunity

**Figure 8:**
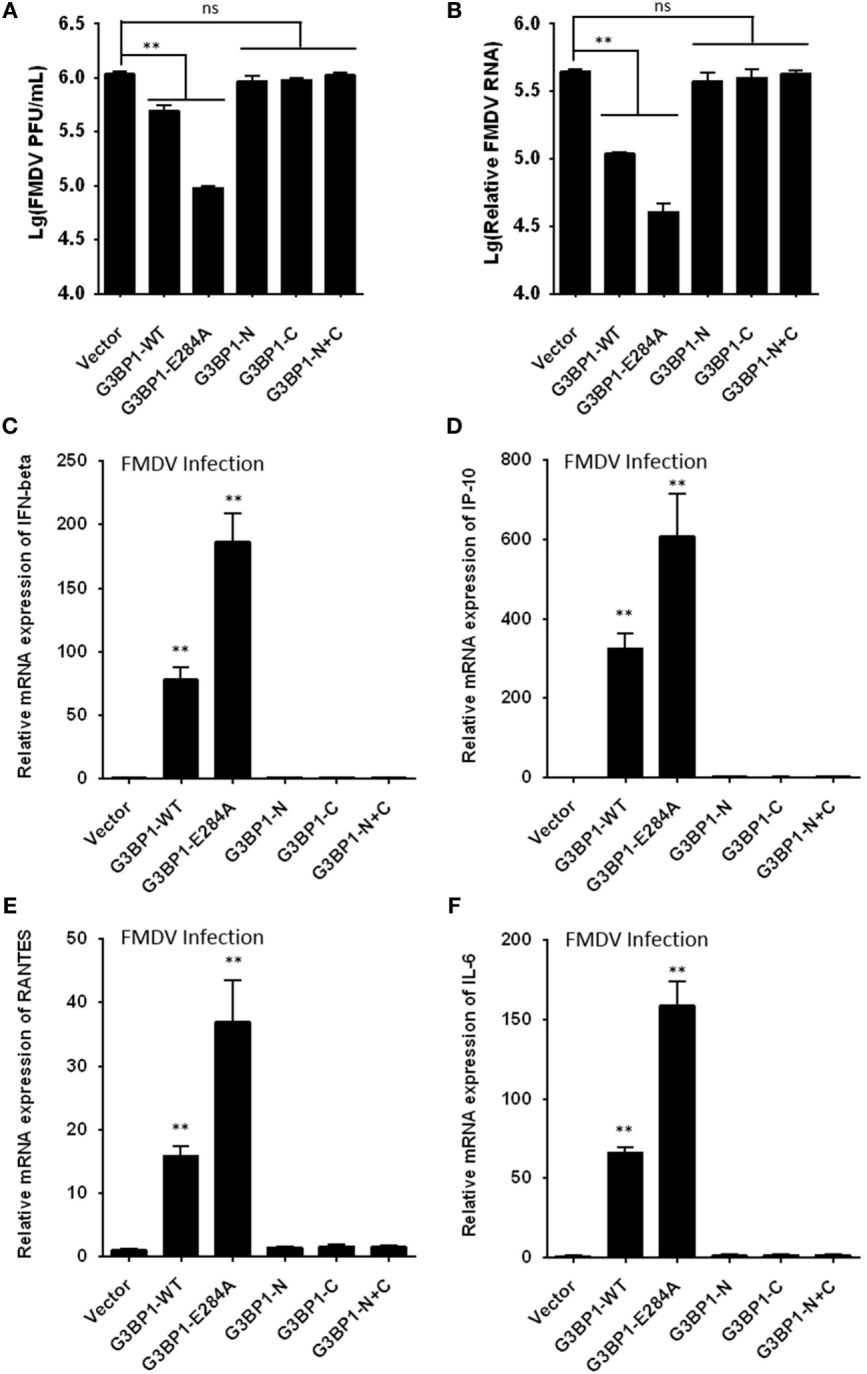
G3BP1 inhibits foot-and-mouth disease virus (FMDV) replication and induces interferon (IFN) and other cytokines expression in FMDV-infected cells. **(A,B)** IBRS-2 cells were transfected with the designated Flag-tagged porcine G3BP1 expression plasmids or an empty vector, and, 30 h later, the cells were infected with FMDV (MOI = 5). 6 h after FMDV infection, virus plaque **(A)** and real-time PCR **(B)** were performed to analyze for virus titer and genome RNA. **(C–F)** IBRS-2 cells were transfected with plasmid encoding Flag-tagged wild-type porcine G3BP1 or its mutants as indicated, and, 30 h later, the cells were infected with FMDV (MOI = 5). 6 h after FMDV infection, total RNA was extracted and the expression of IFN-beta **(C)**, IP-10 **(D)**, RANTES **(E)**, IL-6 **(F)**, and glyceraldehyde-3-phosphate dehydrogenase genes were evaluated by quantitative real-time reverse transcriptase-PCR.

To further determine the impact of 3C^pro^-induced G3BP1 cleavage on FMDV replication, we transfected cells with the different G3BP1 expression plasmids for 24 h, then challenged cells with FMDV. Overexpression of wild-type porcine G3BP1 significantly inhibited FMDV replication as revealed by progeny virus production (Figure 8A) and viral RNA replication (Figure 8B). Interestingly, the E284A mutation, which renders G3BP1 resistant to cleavage by 3C^pro^, enhanced the antiviral activity of G3BP1 (Figures 8A,B). In stark contrast, overexpression of the N- or C-fragment of G3BP1, or in combination, had no appreciable antiviral effect. These data confirm that G3BP1 is a host factor that suppresses FMDV replication and that its antiviral activity depends on the integrity of the full-length protein.

Recent studies have shown that SG formation is an important aspect of the antiviral response (36, 44–49). G3BP1 promotes innate immune responses through NF-κB activation and the PXXP domain in G3BP1 is essential for its antiviral activity (45, 46). Because the 3C^pro^ cleavage site (E284) resides in the PXXP domain, we next investigated whether 3C^pro^-mediated cleavage impairs the antiviral activity of G3BP1. First, we tested whether porcine G3BP1 could induce NF-κB and IFN regulatory factors (IRFs) activation, which are required for the production of type I IFN and many cytokine genes. Consistent with previous work on human G3BP1 (46), overexpression of porcine G3BP1 significantly induced NF-κB activation, but not IRFs activation (Figure S4A in Supplementary Material). However, NF-κB activation was dose-dependently reduced in FMDV-infected cells (Figure S4B in Supplementary Material) and in cells expressing 3C^pro^ alone (Figure S4C in Supplementary Material), suggesting that 3C^pro^ inhibits G3BP1-induced NF-κB-dependent innate immune response. Subsequently, we examined IFN-β and inflammatory cytokine expression in cells infected with FMDV, with or without co-expression of WT G3BP1 or the 3C^pro^-resistant E284A mutant. Compared with control vector, overexpression of WT G3BP1 promoted the induction of IFN-β (Figure 8C), IP-10 (IFN-inducible protein 10, also known as CXCL10) (Figure 8D), RANTES (regulated upon activation, normal T-cell expressed and secreted, also known as CCL5) (Figure 8E), and IL-6 (interleukin 6) (Figure 8F) by FMDV. In comparison, significantly more potent effect was seen with the E284A mutant. Importantly, both 3C^pro^-induced cleavage products of G3BP1 acted like the control vector, i.e., having lost the ability to promote innate immune gene induction following FMDV infection. These results lend further support to the notion that G3BP1 cleavage by 3C^pro^ in FMDV-infected cells dampens the induction of innate antiviral immune responses.

## Discussion

Foot-and-mouth disease is one of the most contagious diseases of cloven-hoofed animals. As with many other positive-strand RNA viruses, the viral genome of FMDV is a functional mRNA that must be efficiently translated as the first step of the infectious cycle (1, 2). Thus, it is of no surprise that FMDV rapidly takes over the cellular translation machinery. Since the host quickly responds to stress and altered environmental conditions, it is crucial for the virus to effectively co-opt multiple aspects of the translation apparatus to not only promote its own gene expression, but also to restrict cellular mechanisms that activate innate immunity (4). FMDV encodes two viral proteinases, L^pro^ and 3C^pro^, that work in concert to rapidly shutdown host cap-dependent translation by cleaving and inactivating eIF4G and eIF4A translation initiation factors (5, 6). To aid in understanding FMDV pathogenesis, in this study we characterized the proteome and phosphoproteome of FMDV-infected cells to elucidate the intracellular signaling pathways and critical host factors regulated by the virus. It was found that ~540 cellular proteins were downregulated (fold change <0.67, *p* < 0.05), while merely 13 cellular proteins were upregulated (fold change >1.5, *p* < 0.05) in FMDV-infected cells at 6 hpi (Figure 1F; Table S3 in Supplementary Material). We note that no detectable cytopathic effects, apoptosis, and cell death by necrosis was observed at this time (Figures 1B,C). In addition, several host translation initiation factors, such as eIF4G, eIF3, and eIF2α, were significantly downregulated as revealed by proteome data (Table S3 in Supplementary Material). These data support that the FMDV shutoff of host cap-dependent translation is mediated by downregulating the expression of translation initiation factors. This mechanism most likely underpins the observation that a large number of proteins in the cellular proteome are downregulated by FMDV.

Foot-and-mouth disease virus IRES elements are highly structured RNA sequences in the 5′ noncoding region that function to recruit ribosomes for the initiation of cap-independent translation of viral polyprotein (2). The IRES comprises several stem loops and pseudoknots and serves to efficiently bind both canonical translation initiation factors and other RBPs (3). Given the ability of RBPs to recognize multiple targets, it is conceivable that secondary protein–protein or RNA–protein bridges could facilitate IRES activity. A prime example of this is Ebp1 (ErbB3-binding protein-1, also known as ITAF45 or PA2G4), which cooperates with PTBP1 to stimulate FMDV IRES activity (22). Although early studies defined numerous ITAFs as proteins that stimulate IRES activity, various RBPs acting as IRES downregulators have been identified in recent studies. For instance, the cytoplasmic protein Gemin5 binds directly to the FMDV IRES, impeding translation (21, 50). However, this protein is cleaved in FMDV-infected cells associated with expression of FMDV L^pro^ (50). Recently, another RBP, G3BP1, was identified as an FMDV IRES-transacting factor by riboproteomic analysis, but functional studies have not been reported (20). Here, we show that overexpression of G3BP1 significantly inhibited FMDV IRES activity, whereas the inhibition was removed in FMDV-infected cells. Our data uncover an unappreciated role of G3BP1 in viral translational control as a negative regulator of FMDV IRES and suggest a novel antiviral mechanism imparted by this RBP.

It has established that picornavirus-mediated control of host mRNA translation involves changes in the phosphorylation status of eIFs (18, 19). Polivirus (PV) infection inhibits cap-dependent translation through inducing eIF2α phosphorylation (19). In addition, eIF4E is targeted in encephalomyocarditis virus (EMCV) and PV-infected cells through virus-induced dephosphorylation of 4E-BPs. Hypophosphorylated 4E-BPs associate strongly with eIF4E, preventing eIF4E from binding to eIF4G, and as a result, inactivating cap-dependent translation (18). Furthermore, G3BP1 is hyperphosphorylated on serine residues in quiescent cells (51), however, our phosphoproteome analysis and immunoblotting data demonstrate that specific dephosphorylation of G3BP1 at Ser-149 occurred in FMDV-infected cells (Table S1 in Supplementary Material; Figure 4C). As recently pointed out by Reineke et al., casein kinase 2 (CK2) is critical for phosphorylations of G3BP1 at Ser-149. Interestingly, the respective peptide of CK2 subunit, β subunits decreased in abundance in FMDV-infected cells (Table S2 in Supplementary Material), suggesting that FMDV may dephosphorylate G3BP1 by CK2 sequestration upon infection (52). Moreover, mutations that prevent the phosphorylations at these residues abrogate the ability of G3BP1 to inhibit FMDV IRES (Figure 4D), arguing that dephosphorylation of G3BP1 induced by FMDV infection is a mechanism by which the virus antagonizes G3BP1-mediated antiviral effect through the IRES.

As discussed above, FMDV infection leads to rapid host translation shutoff through viral cleavage of eIF4G and eIF4A (5, 6). This will produce thousands of idle, stalled host mRNAs. To cope with this stress, host cells assemble SGs to compartmentalize and store the accumulating transcripts (53). Dephosphorylation of G3BP1 at Ser149 has been shown to induce SG assembly after arsenite treatment (30). Since hypophosphorylated G3BP1 at this residue was observed in FMDV-infected cells, one would expect SGs to be induced by FMDV infection. However, FMDV did not trigger SG accumulation in infected cells. This can be explained by our novel finding that 3C^pro^ proteolytically cleaves G3BP1, thereby efficiently inactivating this SGs inducer. Previous work has shown that infection with PV, EMCV, or Coxsackievirus B3 also results in G3BP1 cleavage and disassembly of SGs (34–36). All three viruses target G3BP1 at the same site (Q325), whereas the 3C-like protease of Feline Calicivirus specifically cleaves G3BP1 at E405 (37). Our mutational analysis showed that FMDV 3C^pro^ cleaves G3BP1 at E284 (Figure 7B). To the best of our knowledge, the structural basis for disparate G3BP1 recognition and cleavage by different viral proteases has not been examined to date. Recent evidence suggests that G3BP2, a homolog of G3BP1, also facilitates SG formation, and G3BP1 and G3BP2 exhibit compensatory functions (54). Our proteome and phosphoproteome data show that the protein abundance and phosphorylation of G3BP2 were both downregulated in FMDV-infected cells (Tables S1 and S3 in Supplementary Material). Given that the 3C^pro^ cleavage recognition sequence around E284 in G3BP1 is highly conserved in G3BP2 and that SGs formation was nearly completely inhibited in 3C^pro^-expressing cells, it is quite possible that G3BP2 is also cleaved and inactivated by 3C^pro^ during FMDV infection. This will need to be investigated in future studies.

Accumulating evidence has suggested that connections exist between SGs and innate immune factors (44). It has been reported that SGs induced by G3BP1 overexpression exert an antiviral effect against multiple picornaviruses (35, 36, 46). For example, EMCV induced transient formation of SGs, which were required for efficient activation of IFN and cytokine genes (36). Our data establish that G3BP1 is a novel antiviral factor against FMDV, not only by suppressing IRES-mediated viral protein expression, but also by inducing antiviral gene expression (Figures 8C–F). Significantly, a 3C^pro^-resistant G3BP1 mutant, E284A was more effective than the WT protein in suppressing viral replication and inducing innate immune activation, underscoring the importance of the 3C^pro^–G3BP1 axis in tipping the balance between FMDV and host cells.

Apart from G3BP1’s role in inducing antiviral gene expression, assembly of large G3BP1-induced SGs causes PKR (protein kinase R, also known as EIF2AK2) activation that leads to NF-κB activation. PKR is also an important signaling molecule implicated in innate immune activation (46). Since FMDV disrupts SGs by cleaving G3BP1, G3BP1-dependent PKR activation and subsequent cytokine induction could be impaired. Supporting this notion, dephosphorylation of PKR was observed in FMDV-infected cells by our phosphoproteome analysis (Table S1 in Supplementary Material).

It should be noted that although a G3BP1 mutant that cannot be phosphorylated (S149A) impaired the ability to suppress FMDV IRES (Figure 4D), and both the N-terminal and C-terminal G3BP1 cleavage products of 3C^pro^ were incapable of FMDV IRES inhibition (Figure 7F), SG assembly (Figure S3B in Supplementary Material), and innate immune activation (Figures 8C–F), no complete dephosphorylation or cleavage of G3BP1 was observed following FMDV infection in our experiments (Figures 4C and 5B). It is possible that the viral targeting of G3BP1 may be more complete at a later stage of FMDV infection (e.g., beyond 9 hpi). Not mutually exclusive, FMDV-induced dephosphorylation and cleavage of G3BP1 may be operated in concert to maximally cripple the multiple functions of G3BP1 and provide FMDV a survival advantage.

In summary, our study unveils G3BP1 as a novel antiviral host factor against FMDV, which operates by inhibiting FMDV IRES-dependent translation and activating innate immune responses. FMDV, in turn, has evolved to limit this innate defense mechanism by inducing dephosphorylation and proteolytic cleavage of G3BP1. These revelations add a new layer of complexity to the immune evasion tactics devised by this economically important viral pathogen.

## Author Contributions

SX, DW, LF, and KL designed research. TP, XY, JM, XZ, YS, and DW performed research. DW, SX, KL, XY, TP, and JM analyzed data. DW, SX, KL, and TP wrote the manuscript. SX, HZ, KZ, LF, and HC contributed new reagents/analytic tools. All the authors read and approved the manuscript.

## Conflict of Interest Statement

The authors declare that the research was conducted in the absence of any commercial or financial relationships that could be construed as a potential conflict of interest.
