# Corrigendum: Foot-and-Mouth Disease Virus Counteracts on Internal Ribosome Entry Site Suppression by G3BP1 and Inhibits G3BP1-Mediated Stress Granule Assembly *via* Post-Translational Mechanisms

**DOI:** 10.3389/fimmu.2021.702530

**Published:** 2021-11-04

**Authors:** Xu Ye, Ting Pan, Dang Wang, Liurong Fang, Jun Ma, Xinyu Zhu, Yanling Shi, Keshan Zhang, Haixue Zheng, Huanchun Chen, Kui Li, Shaobo Xiao

**Affiliations:** ^1^ State Key Laboratory of Agricultural Microbiology, College of Veterinary Medicine, Huazhong Agricultural University, Wuhan, China; ^2^ The Cooperative Innovation Center for Sustainable Pig Production, Wuhan, China; ^3^ National Foot and Mouth Diseases Reference Laboratory, Lanzhou Veterinary Research Institute, Chinese Academy of Agricultural Sciences, Lanzhou, China; ^4^ Department of Microbiology, Immunology and Biochemistry, University of Tennessee Health Science Center, Memphis, TN, United States

**Keywords:** foot-and-mouth disease virus, phosphoproteomics, G3BP stress granule assembly factor 1, internal ribosome entry site, innate immunity

In the original article, there was a mistake in **Figure 6A** as published. We checked the raw data and found that we accidentally cropped the control group, so there is a control group missing from the Western blot experiment for detecting Flag-G3BP1. Lane 1 was used to show that the specific bands detected by Western blot were the ectopic expression of Flag-G3BP1, as the same background/noise was present in lane 1 as in lanes 2-6 (compared to blank lanes), but no specific bands were detected in lane 1. The corrected **Figure 6** appears below.

**Figure 6:**
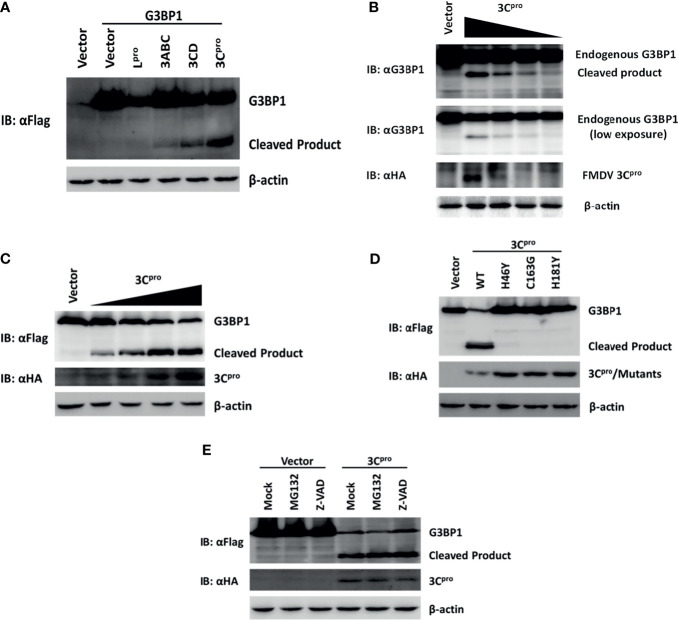
Foot-and-mouth disease virus 3C^pro^ cleaves G3BP1 by means of its protease activity. **(A)** Human embryonic kidney cells (HEK)-293T cells cultured in 60-mm dishes were transfected with Flag-tagged porcine G3BP1 as indicated (4 μg), along with HA-L^pro^, 3C^pro^, or 3C^pro^-containing precursors (0.05 μg). Cell lysates were prepared 30 h post-transfection and analyzed by western blotting. **(B)** IBRS-2 cells were transfected with increasing quantities (0, 0.5, 1, 2, or 4 μg) of plasmid encoding 3C^pro^. Cell lysates were prepared 36 h post-transfection and analyzed by western blotting. **(C)** HEK-293T cells were transfected with Flag-tagged wild-type porcine G3BP1 (4 μg), along with increasing quantities HA-3C^pro^ plasmid (0, 0.0125, 0.025, 0.05, or 0.1 μg). Cell lysates were prepared 30 h post-transfection and analyzed by western blotting. **(D)** HEK-293T cells were transfected with Flag-tagged porcine G3BP1 expression plasmid (4 μg), along with wild-type 3C^pro^ expression plasmids or its mutants (0.05 μg). Cell lysates were prepared 30 h post-transfection and analyzed by western blotting. **(E)** HEK-293T cells were co-transfected with Flag-tagged porcine G3BP1 expression plasmid (4 μg) and plasmid encoding 3C^pro^ or empty vector (0.05 μg). 24 h after transfection, MG132 or zVAD-FMK were added to a final concentration of 20 μM. Cell lysates were prepared 8 h after treatment and analyzed by western blotting.

The authors apologize for this error and state that this does not change the scientific conclusions of the article in any way. The original article has been updated.

## Publisher’s Note

All claims expressed in this article are solely those of the authors and do not necessarily represent those of their affiliated organizations, or those of the publisher, the editors and the reviewers. Any product that may be evaluated in this article, or claim that may be made by its manufacturer, is not guaranteed or endorsed by the publisher.

