# Supplementary material for: Foot-and-Mouth Disease Virus Counteracts on Internal Ribosome Entry Site Suppression by G3BP1 and Inhibits G3BP1-Mediated Stress Granule Assembly via Post-Translational Mechanisms
Source: Front Immunol. 2018 May 25;9:1142. doi: 10.3389/fimmu.2018.01142 (PMC5980976; doi:10.3389/fimmu.2018.01142)
Supplement: Supplementary file 1 [file Presentation_1.zip › Supplementary Material Presentation/Figure S1.pdf]

Figure S1

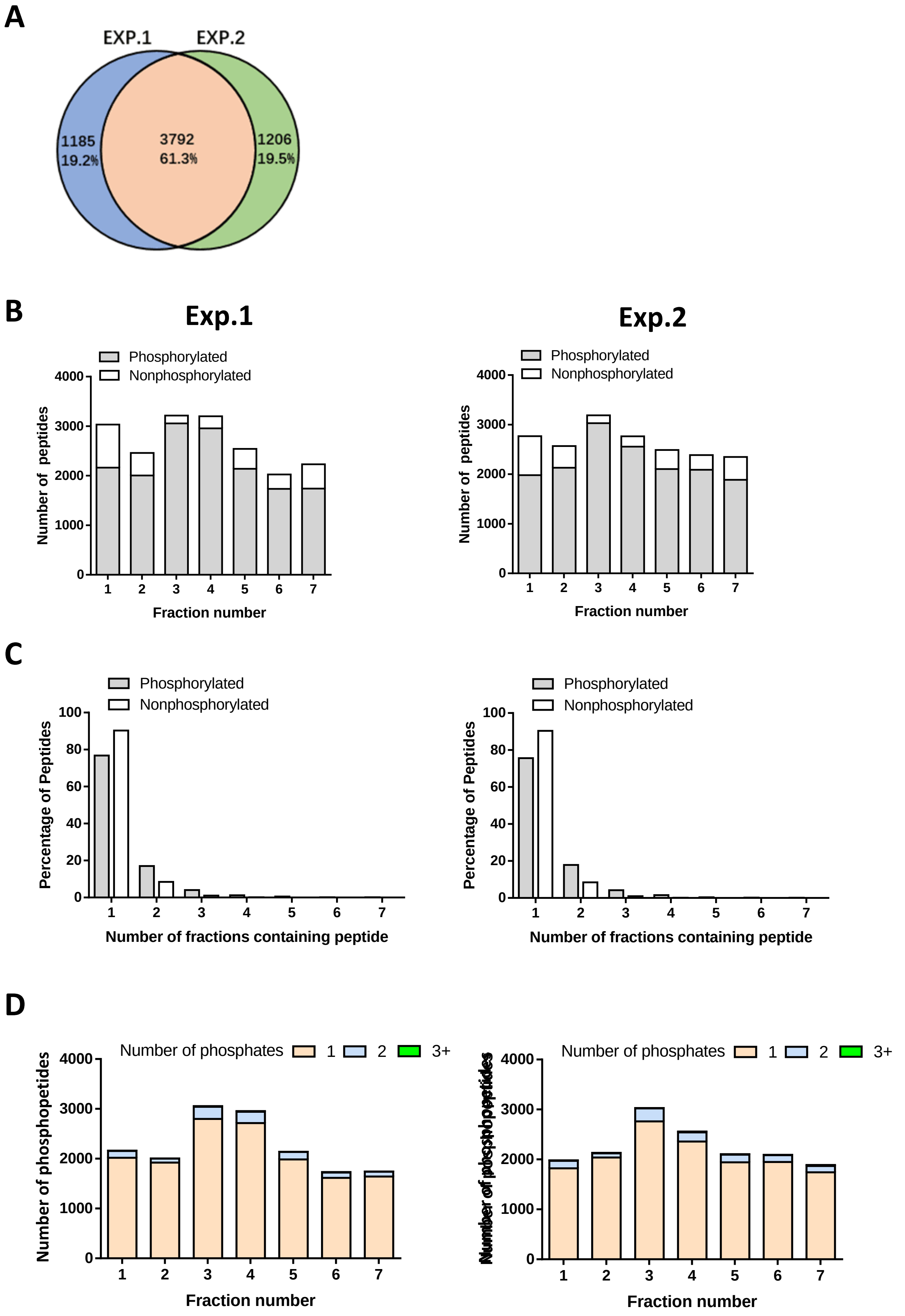
A
EXP.1
EXP.2
1185
19.2%
3792
61.3%
1206
19.5%
B
Exp.1
Exp.2
Phosphorylated
Nonphosphorylated
Number of peptides
Fraction number
C
Phosphorylated
Nonphosphorylated
Percentage of Peptides
Number of fractions containing peptide
D
Number of phosphates
1
2
3+
Number of phosphopeptides
Fraction number

Figure S1. Phosphoproteomics analysis of FMDV-infected IBRS-2 cells in the two replicate experiments. (**A**) Area-proportional Euler diagram showing the overlap of phosphosites identified in the two replicate experiments. (**B**) Bar chart indicating the unique phosphorylated and nonphosphorylated peptides identified in each HPLC fraction. Numbers report the sum of peptides identified in two replicate experiments, respectively. (**C**) Bar chart showing the number of HPLC fractions in which individual phosphopeptides and nonphosphopeptides were detected in two replicate experiments. (**D**) Bar chart showing the number of phosphorylated residues on phosphopeptides in each HPLC fraction. Numbers report the sum of phosphopeptides in two replicate experiments.
