# Supplementary material for: Foot-and-Mouth Disease Virus Counteracts on Internal Ribosome Entry Site Suppression by G3BP1 and Inhibits G3BP1-Mediated Stress Granule Assembly via Post-Translational Mechanisms
Source: Front Immunol. 2018 May 25;9:1142. doi: 10.3389/fimmu.2018.01142 (PMC5980976; doi:10.3389/fimmu.2018.01142)
Supplement: Supplementary file 1 [file Presentation_1.zip › Supplementary Material Presentation/Figure S2.pdf]

Figure S2

A

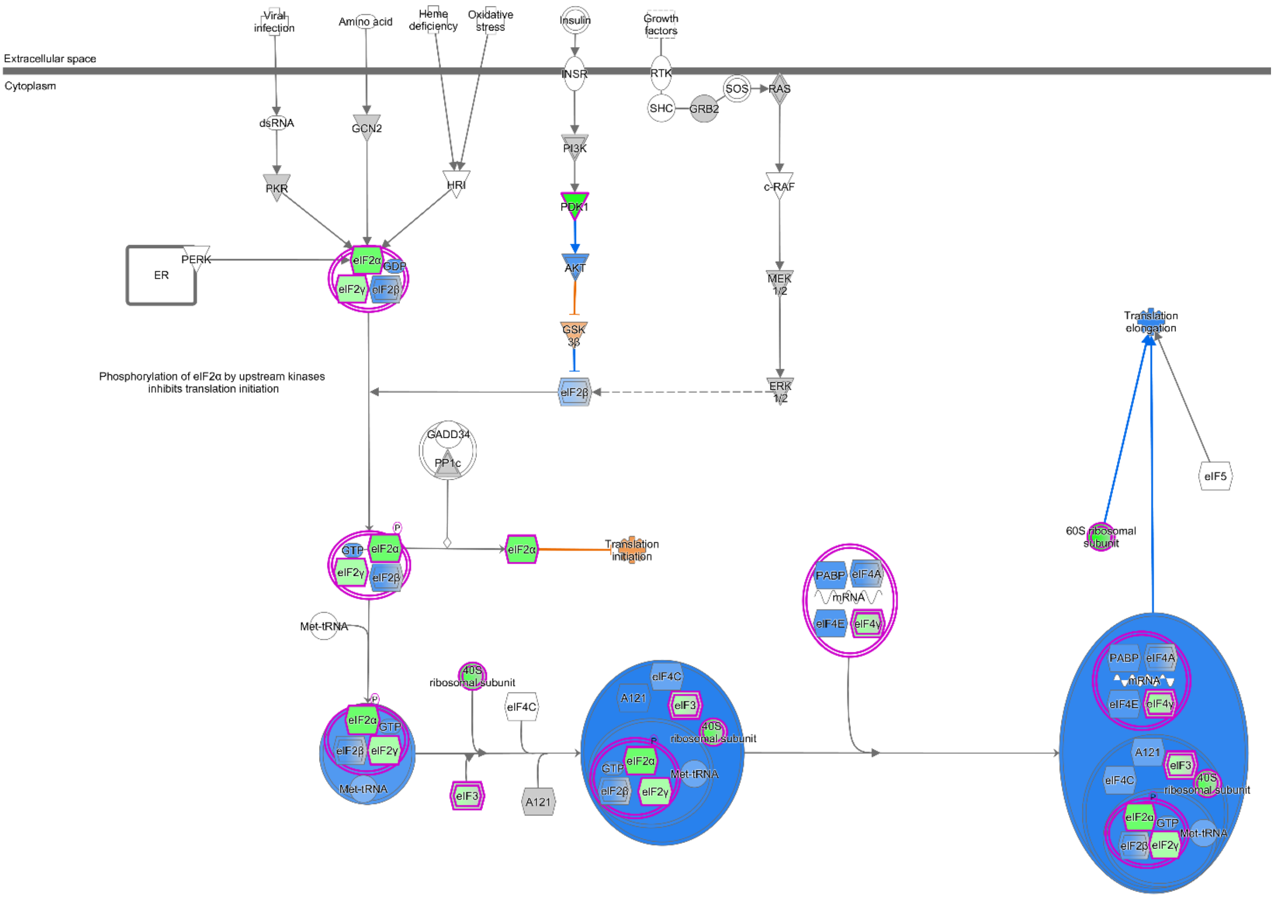

B

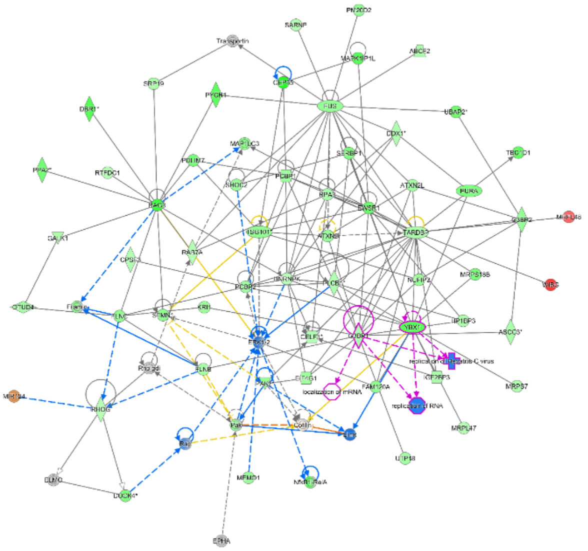

C

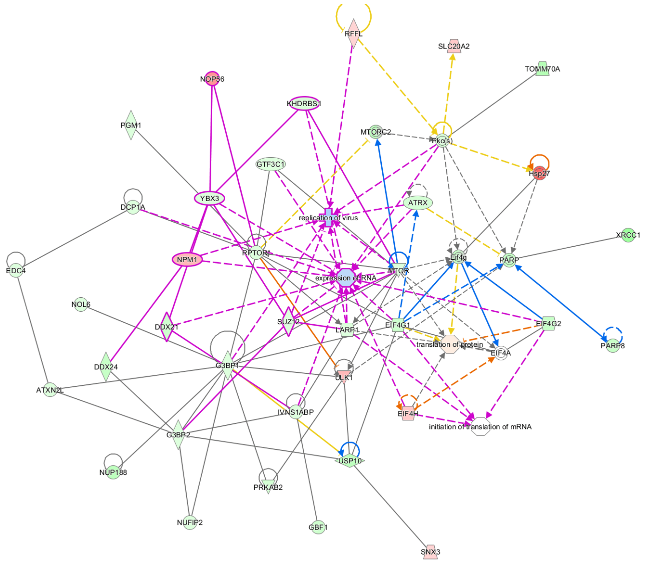

Figure S2. Analysis of proteins significantly altered in FMDV-infected cells by Ingenuity pathway analysis (IPA®). (**A**) Pathway involved in host cap-dependent gene expression is depicted by IPA. Green indicates decreased protein abundance in FMDV-infected IBRS-2 cells, whereas gray shows the unregulated proteins. The proteins corresponding to the predicted downregulated pathway are shown in blue. (**B-C**) IPA-based network of G3BP1 and the target molecules in the proteome and phosphoproteome data. Green indicates decreased protein or phosphorylation in FMDV- infected IBRS-2 cells, whereas red shows increased in FMDV-infected IBRS-2 cells. Dashed lines represent indirect interactions.
