# Supplementary material for: Foot-and-Mouth Disease Virus Counteracts on Internal Ribosome Entry Site Suppression by G3BP1 and Inhibits G3BP1-Mediated Stress Granule Assembly via Post-Translational Mechanisms
Source: Front Immunol. 2018 May 25;9:1142. doi: 10.3389/fimmu.2018.01142 (PMC5980976; doi:10.3389/fimmu.2018.01142)
Supplement: Supplementary file 1 [file Presentation_1.zip › Supplementary Material Presentation/Figure S3.pdf]

Figure S3

A

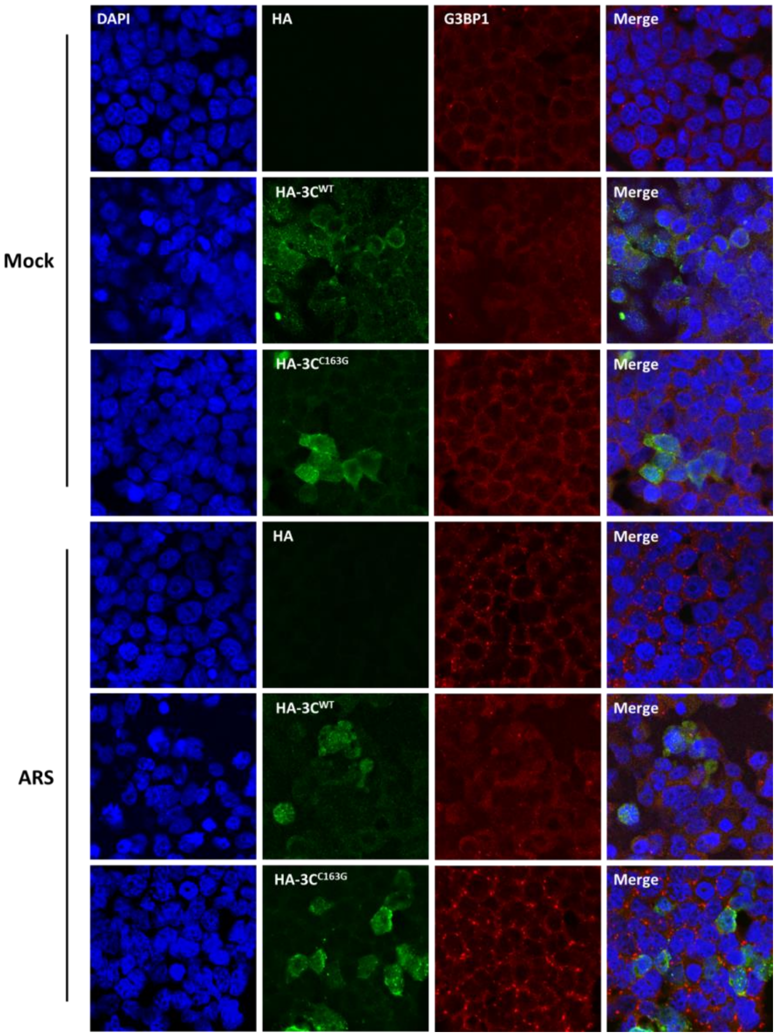
DAPI
HA
G3BP1
Merge
Mock
HA-3C^WT
Merge
HA-3C^C163G
Merge
HA
Merge
ARS
HA-3C^WT
Merge
HA-3C^C163G
Merge

B

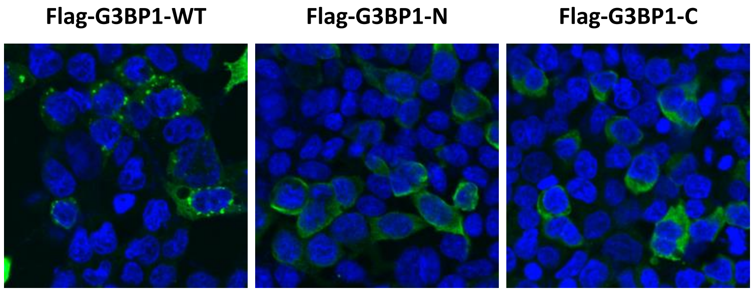
Flag-G3BP1-WT
Flag-G3BP1-N
Flag-G3BP1-C

Figure S3. FMDV 3Cpro-induced G3BP1 cleavage fragments exhibit impaired SGs assembly. (**A**) HEK-293T cells were transfected with the designated HA-tagged 3Cpro expression plasmids or an empty vector, and, 30 h later, the cells were treated with arsenite. One hour after the treatment, indirect immunofluorescence assays were performed. The cells were fixed and incubated with a monoclonal antibody against HA tag (green) and a polyclonal antibody against G3BP1 (red). The nuclei of cells were stained with DAPI (blue). (**B**) HEK-293T cells were transfected with the designated flag-tagged porcine G3BP1 expression plasmids, and, 30 h later, the cells were treated with arsenite. One hour after the treatment, indirect immunofluorescence assays were performed. The cells were fixed and incubated with a monoclonal antibody against Flag tag (green) and the nuclei of cells were stained with DAPI (blue).
