# Supplementary material for: Foot-and-Mouth Disease Virus Counteracts on Internal Ribosome Entry Site Suppression by G3BP1 and Inhibits G3BP1-Mediated Stress Granule Assembly via Post-Translational Mechanisms
Source: Front Immunol. 2018 May 25;9:1142. doi: 10.3389/fimmu.2018.01142 (PMC5980976; doi:10.3389/fimmu.2018.01142)
Supplement: Supplementary file 1 [file Presentation_1.zip › Supplementary Material Presentation/Figure S4.pdf]

Figure S4

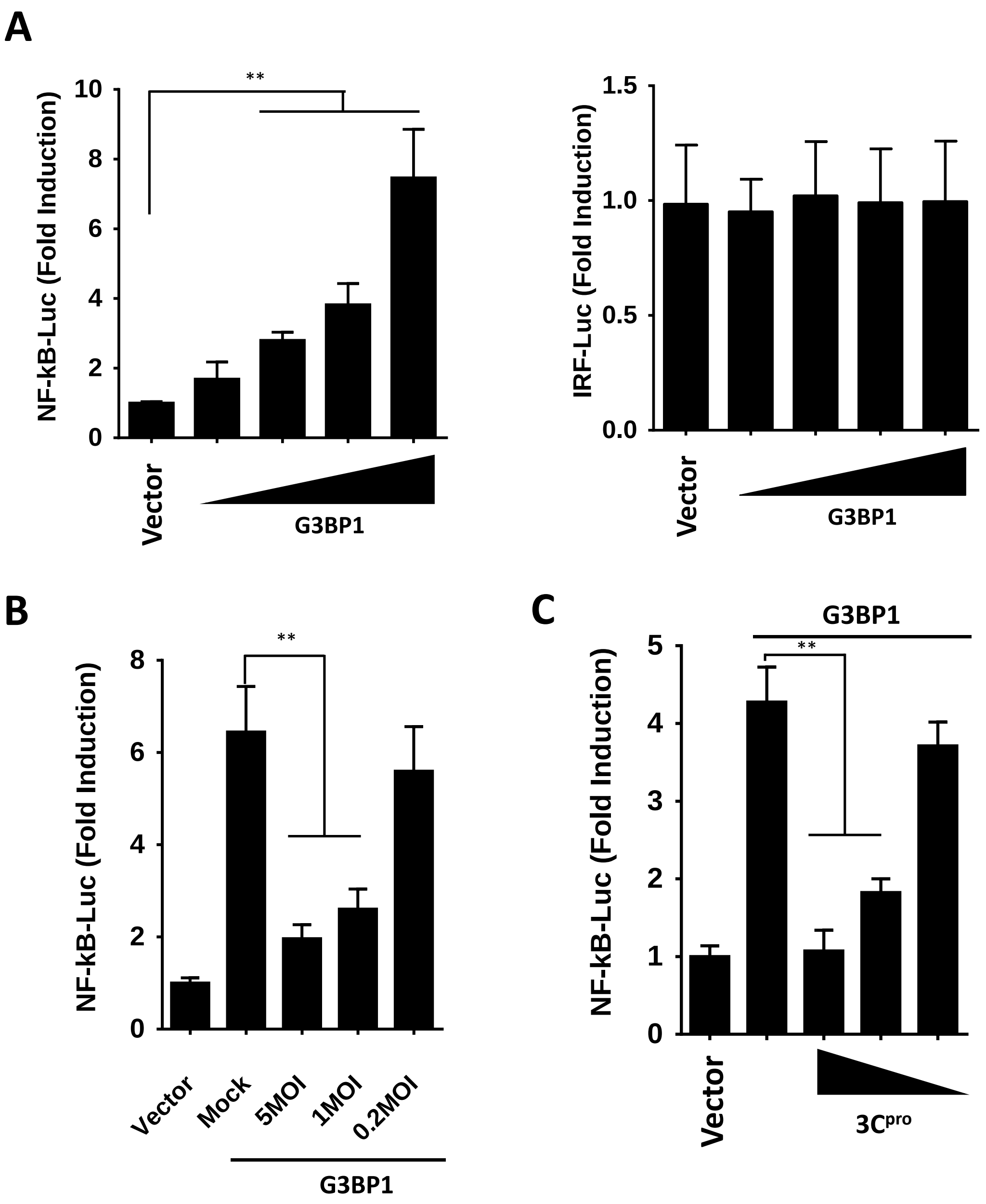

Figure S4. FMDV 3Cpro inhibits G3BP1-induced NF-κB-dependent innate immune response. (A) IBRS-2 cells were transfected with NF-κB-Luc/IRFs-Luc, and pRL-TK plasmid (Promega) (for normalization of transfection efficiency) along with increasing quantities of plasmid encoding porcine G3BP1. Luciferase assays were performed 36 h after transfection. (B) IBRS-2 cells were transfected with NF-κB-Luc, and pRL-TK plasmid along with porcine G3BP1 expression plasmid or an empty vector (0.5 μg). 30 h after initial transfection, cells were infected with FMDV at different MOIs. Luciferase assays were performed 6 h after FMDV infection. (C) IBRS-2 cells were transfected with NF-κB-Luc, pRL-TK plasmid, and Flag-tagged porcine G3BP1 expression plasmid, along with increasing quantities of the plasmid encoding 3Cpro. Luciferase assays were performed at 36 h after the transfection.
